# How to add baskets to an ongoing basket trial with information borrowing

**DOI:** 10.1177/09622802251316961

**Published:** 2025-03-20

**Authors:** Libby Daniells, Pavel Mozgunov, Helen Barnett, Alun Bedding, Thomas Jaki

**Affiliations:** 1 STOR-i Centre for Doctoral Training, Department of Mathematics and Statistics, 4396 Lancaster University, Lancaster, UK; 2 MRC Biostatistics Unit, University of Cambridge, Cambridge, UK; 3 School of Mathematical Sciences, 4396 Lancaster University, Lancaster, UK; 4 Roche Products Ltd, Welwyn Garden City, UK; 5 Faculty of Informatics and Data Science, 9147 University of Regensburg, Regensburg, Germany

**Keywords:** Basket trial, adaptive design, calibration, information borrowing, Bayesian modelling, error control

## Abstract

Basket trials test a single therapeutic treatment on several patient populations under one master protocol. A desirable adaptive design feature is the ability to incorporate new baskets to an ongoing trial. Limited basket sample sizes can result in reduced power and precision of treatment effect estimates, which could be amplified in added baskets due to the shorter recruitment time. While various Bayesian information borrowing techniques have been introduced to tackle the issue of small sample sizes, the impact of including new baskets into the borrowing model has yet to be investigated. We explore approaches for adding baskets to an ongoing trial under information borrowing. Basket trials have pre-defined efficacy criteria to determine whether the treatment is effective for patients in each basket. The efficacy criteria are often calibrated a-priori in order to control the basket-wise type I error rate to a nominal level. Traditionally, this is done under a null scenario in which the treatment is ineffective in all baskets, however, we show that calibrating under this scenario alone will not guarantee error control under alternative scenarios. We propose a novel calibration approach that is more robust to false decision making. Simulation studies are conducted to assess the performance of the approaches for adding a basket, which is monitored through type I error rate control and power. The results display a substantial improvement in power for a new basket, however, this comes with potential inflation of error rates. We show that this can be reduced under the proposed calibration procedure.

## 1. Introduction

Basket trials are a form of master protocol in which a single treatment is administered to patients across different disease types, all of whom possess the same genetic aberration. Different disease type sub-populations form their own treatment basket.^1^ Typically, basket trials are implemented in the early stages of the drug development process in order to determine the efficacy of a treatment in each of the individual baskets on the trial.^2^ They often consist of a single treatment arm using a small number of patients.

One of the main benefits of basket trials is that they allow testing of treatments on small sub-groups of patients, which may result from being in the early-phase setting or from investigating rare diseases.^3^ With such small sample sizes, individual studies for each condition would not traditionally be warranted due to financial and time constraints. By allowing for testing on multiple disease types in a single study, the drug development process is substantially expedited.^4,5^ Basket trials, like other efficient study designs such as platform and umbrella trials, can provide flexibility by utilising adaptive design features, which allow for modification of the design and analysis while the study is still ongoing. Such modifications include interim analysis with futility and efficacy stopping, sample size adjustment, or as is the focus of this work, the addition of a single or multiple baskets to an ongoing trial. This situation could arise when a new group of patients is identified to potentially benefit from the treatment, where these patients harbour the genetic aberration under investigation, but suffer from a different type of disease.

Several prominent clinical trials have utilised the addition of a basket. An example of this is the VE-BASKET trial,^6^ exploring the effect of vemurafeib on various non-melanoma cancers with the BRAFV600 mutation. In this study, the number of baskets comprising the study changed while the trial was ongoing. The study opened with six disease-specific baskets, three of which were closed due to insufficient accrual. Two baskets were added due to sufficient enrolment of patients in an ‘all other’ basket consisting of patients with BRAFV600 mutations but with different disease types to the defined baskets. In addition to the VE-BASKET trial, there is an ongoing basket trial that is looking at the effect of tucatinib and trastuzumab on a number of solid tumours with the HER2 alteration.^7^ The established baskets include cervical cancer, uterine cancer, urothelial cancer amongst others, and like the VE-BASKET trial, this trial also included two baskets consisting of all other HER2 amplified solid tumour types or HER2 mutated solid tumour types.^8^ The study protocol of the HER2 trial outlines the ability to adapt the trial design based on recruitment rates within the two ‘all other’ baskets, which will allow new disease-specific baskets to be formed within the trial. Both the VE-BASKET and HER2 trials feature the addition of baskets within their trial protocol, however, it is not stated explicitly how these new baskets are analysed compared to baskets that began the trial. In both trials it appears stratified analysis of each basket is (or will) be conducted, thus these new baskets being formed will have no impact on the established baskets on the trial. This is with the exception of the ‘all other’ basket, where the sample size was reduced as the new baskets were created from the patients within this basket. Should information be shared between the established and new baskets, the added baskets will have an impact on inference in all baskets on the trial. Thus, when information is shared, careful consideration on how to handle the addition of baskets is required. This motivates the work presented in this paper, with the purpose of exploring methodology for analysing trials where baskets have been added.

While basket trials are desirable as they allow the testing of treatments on small groups of patients, a prominent issue in basket trials is the lack of statistical power and precision of estimates. This can be amplified in baskets that are added part-way through an ongoing trial. The combination of reduced recruitment rate (when the new disease type is rare) and shorter recruitment time due to the late addition to the trial, can result in a further reduction in sample sizes compared to baskets that opened at the beginning of the trial. To tackle the issue of small sample sizes, Bayesian information borrowing methods were proposed for use in basket trials. These methods utilise the assumption that, as patients across baskets share the same genetic mutation, they will have a similar response to the treatment. As such, patients are ‘exchangeable’ between baskets, meaning patients can be moved between treatment baskets without changing the overall treatment effect estimates.^9^ One can use this assumption to draw on information from one basket when making inference in another, which has the potential to improve power and precision of estimates. However, when the exchangeability assumption is violated, and there is heterogeneity amongst the response rates in different baskets, any information borrowing has the potential to inflate the type I error rate.^10^ The trade-off between power improvement and error rate inflation among heterogeneous baskets is a well known issue and has been observed in several simulation studies including that by Chu and Yuan,^3^ Jin et al.^11^ and Daniells et al.^12^

Over recent years, several prominent methods for information borrowing in basket trials have been proposed. These include the Bayesian hierarchical model (BHM)^13^ and several adaptations to this method, such as the calibrated Bayesian hierarchical model (CBHM)^3^ which defines the prior on the borrowing parameter as a function of homogeneity, the exchangeability–nonexchangeability model (EXNEX)^14^ which allows for flexible borrowing between subsets of baskets and the modified exchangeability-nonexchangeability model (mEXNEX ${}_{c}$ )^12^ which modifies the EXNEX model to account for homogeneity/heterogeneity between baskets. However, to the best of our knowledge, any discussion on the addition of baskets whilst utilising information borrowing is sparse.

This purpose of this work is to propose and investigate several approaches for the analysis of newly added baskets under an information borrowing structure, which primarily utilises the EXNEX model. To identify when and which approach is deemed appropriate for use, thorough simulation studies under a variety of settings have been conducted, primarily monitoring the type I error rate and power. The simplest approach to such an addition would be to analyse the new baskets akin to baskets that were already in the trial at the start, a problem which is mathematically equivalent to a case of unequal sample sizes. This work also explores additional methodology, which is motivated by the concern that new baskets could negatively impact the type I error rate and power of existing baskets should the response rates be heterogeneous across baskets. However, substantial power can be gained by borrowing from new baskets in cases of homogeneity. Control of the type I error rate in the the new basket must also be considered.

The second novel aspect of this work regards the calibration of efficacy criteria. When implementing Bayesian borrowing models, posterior probabilities are computed and compared to some pre-defined cut-off value in order to determine whether or not a treatment is efficacious in each of the baskets. Traditionally, these cut-off values are calibrated through simulation studies under a global null scenario, where all baskets are truly ineffective. This calibration aims to control the basket specific type I error rate to a nominal level. However, when the cut-off value is applied to cases where at least one basket is effective to treatment, it is not guaranteed that error rates will remain controlled at the nominal level when information borrowing is utilised.^10^ In fact, inflation in the type I error rate often occurs in cases of heterogeneity amongst the response rates across baskets, as borrowing information causes shifts in the posterior probabilities away from the true treatment effect. This brings into question whether calibrating under the global null is sufficient, as more often than not, there is an expectation that the treatment is efficacious in at least one basket. In this work we propose a novel calibration technique, called the **R**obust **Ca**libration **P**rocedure (RCaP), which controls the type I error rate *on average* across several possible true response rate scenarios, with the potential to weight scenarios based on their importance (type I error rate control may be deemed more important under a particular stetting) or the prior likelihood of the scenario occurring in the trial, both of which would be specified by the clinician. Presented in this work is a comparison between operating characteristics under the traditional approach of calibrating under the global null and under the RCaP.

This work is structured as follows, we begin with providing further details on the previously introduced VE-BASKET study. We then describe the EXNEX model, approaches for the analysis of newly added baskets, and outline the novel calibration procedure, RCaP. Results of several simulation studies are presented starting with a comparison of calibration techniques, followed by results of simulation studies to compare performance of the approaches for adding newly identified baskets.

### 1.1. Motivating trial: The VE-BASKET study

The VE-BASKET trial was a phase II, non-randomised, basket trial, investigating the effect of vemurafenib on several cancer types with patients possessing the BRAFV600 mutation.^6^ A total of 122 patients were enrolled across seven baskets, with efficacy evaluated after eight weeks of treatment. The primary endpoint was the overall response rate with a null response rate of 15% indicating inactivity and target response rate of 45%. A response rate of 35% was considered low but still indicative of a response. Sample sizes of 13 patients per basket were obtained through a Simon’s two stage design^15^ based on 80% power and 10% type I error rate.

**Figure 1.**
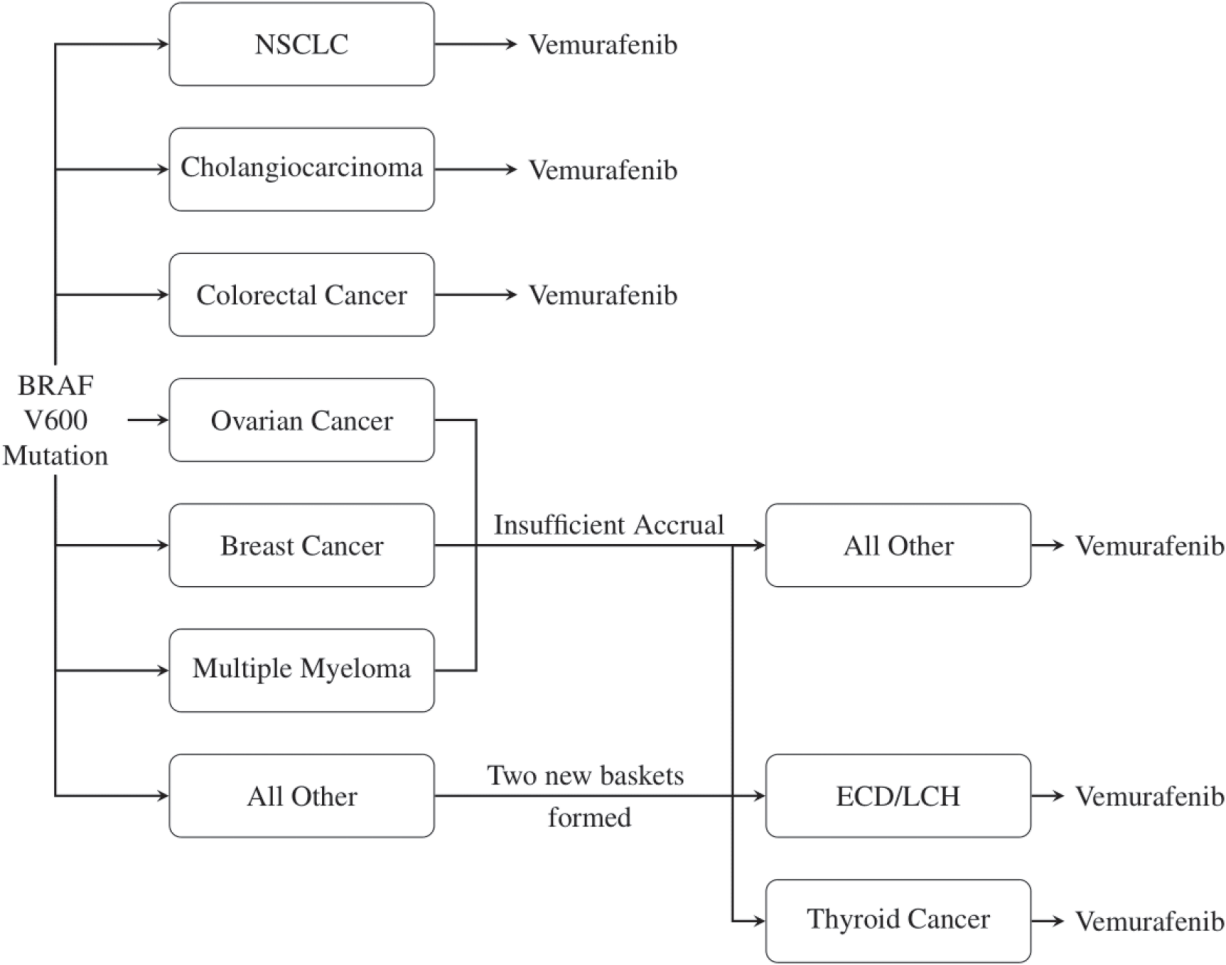
VE-BASKET Trial Design. Vemurafenib is tested on several cancer types, with two new baskets formed from the ‘all other’ group in the trial.

The trial opened with six disease specific baskets: Non-small-cell lung cancer (NSCLC), ovarian cancer, colorectal cancer, cholangiocarcinoma, breast cancer and multiple myeloma. Also present was an ‘all other’ basket consisting of patients with other disease types with the BRAFV600 mutation. This initial trial structure was adapted based on recruitment rates, with the breast cancer, ovarian cancer and multiple myeloma baskets closing due to insufficient accrual. Patients were moved from these baskets to the ‘all other’ basket for analysis. During the trial it was observed that the recruitment of two disease-types in the ‘all other’ basket was high enough to meet the specified sample size requirements for a basket, and thus two new baskets were formed and added to the trial: An Edrheim-Chester disease or Langerhans’ cell histiocytosis (ECD/LCH) basket and an anaplastic thyroid cancer basket. Figure 1 displays the general trial schematic.

The flexible nature of the VE-BASKET trial, with its formation of new baskets and closure of existing ones, brings about the question of how to conduct analysis when these adaptations to the trial design have been made.

## 2. Methodology

### 2.1. Setting

This work focuses on a setting with a single treatment arm within each basket and binary endpoints, in which a patient either responds positively to a treatment or does not. Consider a basket trial with a total of K baskets. Denote the number of responses in basket k by $Y_{k}$ , which follows a binomial distribution, $Y_{k}∼\text{Binomial}(n_{k},p_{k})$ , with $n_{k}$ and $p_{k}$ indicating the sample size and response rate in basket k . Interest lies in estimating the unknown response rate $p_{k}$ . Denote $q_{0}$ and $q_{1}$ as the null and target response rate, respectively.

Now consider a case where baskets of patients are added to an ongoing trial and thus split the K baskets into two sets. Let $K_{0}$ be the total number of baskets that began the trial, labelled as *existing baskets,* thus having $K^{\prime }=K-K_{0}$ *new baskets* added part way through the study. Existing baskets are indexed $k_{0}=1,\ldots ,K_{0}$ and new baskets $k^{\prime }=K_{0}+1,\ldots ,K$ . Note that a new basket, $k^{\prime }$ , may be added at any time during the study and it is not required that all new baskets be added at the same time.

The objective is to test the family of hypotheses:

$$\begin{array}{rlrl} & H_{0}\;:\;p_{k_{0}}\leq q_{0}\;vs.\;H_{a}\;:\;p_{k_{0}}>q_{0}, & k_{0} & =1,\ldots ,K_{0},\\ & H_{0}\;:\;p_{k^{\prime }}\leq q_{0}\;vs.\;H_{a}\;:\;p_{k^{\prime }}>q_{0}, & k^{\prime } & =K_{0}+1,\ldots ,K. \end{array}$$

To test these hypotheses, a Bayesian framework is utilised. Posterior probabilities are used to determine the efficacy of the treatment on each of the individual baskets in the trial. As such, given observed response data D , the treatment is deemed effective in an existing basket $k_{0}$ if $P(p_{k_{0}}>q_{0}|D)>\Delta_{k_{0}}$ and effective in a new basket $k^{\prime }$ if $P(p_{k^{\prime }}>q_{0}|D)>\Delta_{k^{\prime }}$ . Both cut-off values $\Delta_{k_{0}}$ and $\Delta_{k^{\prime }}$ are typically determined through *calibration* in order to control some metric, often related to false decision making, at a nominal level. Traditionally this calibration is done under a global null scenario in which all baskets are ineffective to treatment, in order to control the basket-specific type I error rate to a nominal level.^11,16,17^

### 2.2. The exchangeability--nonexchangeability model

Information borrowing models utilise the exchangeability assumption, which states that as patients across all baskets share a common genetic component, their response to treatment will be similar. Thus information can be shared between baskets in order to improve inference. The BHM first outlined by Berry et al.^13^ is a key basis for many information borrowing models, one of which is the EXNEX model proposed by Neuenschwander et al.^14^ The EXNEX model consists of two components:

1. EX (exchangeable component): With prior probability $\pi_{k}$ , basket k is exchangeable and a BHM is applied. Information borrowing is therefore conducted between all baskets assigned to the exchangeable component.
2. NEX (nonexchangeable component): With prior probability $1-\pi_{k}$ , basket k is nonexchangeable with any other basket, and as a result is analysed independently.
(1) $$\begin{array}{rl} & Y_{k}∼\text{Binomial}(n_{k},p_{k}),\;k=1,\ldots ,K\;M_{1k}∼\text{N}(\mu ,\sigma^{2}),\;\text{(EX)}\\ & \theta_{k}=\text{logit}(p_{k}),\;\;\;\;\;\;\mu ∼\text{N}(\text{logit}(q_{0}),\nu_{\mu }^{2}),\\ & \theta_{k}=\delta_{k}M_{1k}+(1-\delta_{k})M_{2k},\;\;\;\sigma ∼g(\cdot ),\\ & \delta_{k}∼\text{Bernoulli}(\pi_{k}),\;\;\;\;\;M_{2k}∼\text{N}(m_{k},\nu_{k}^{2}).\;\text{(NEX)} \end{array}$$

The EX component has the form of a BHM with the log-odds of the response rates for each basket following a normal distribution, centred around a common mean μ with variance $\sigma^{2}$ . Borrowing occurs between baskets in the EX component where estimates of response rates are shrunk towards the common mean μ with the degree of shrinkage controlled by $\sigma^{2}$ . As $\sigma^{2}$ tends to zero, borrowing moves towards complete pooling of results, however, as it tends to infinity a stratified analysis is conducted on each basket. The prior on μ is centred around the average null response rate across the baskets with a large variance, whilst the prior on σ , g(⋅) , is more widely debated with inverse-gamma, half-normal or half-Cauchy priors implemented across the literature.^18^ In the EXNEX model, Neuenschwander et al.^14^ implement a half-normal(0,1) prior placed on σ as they state that this is a ‘rather conservative prior for the borrowing parameter’ and as such allows for anywhere between a small and large amount of heterogeneity between baskets.

Issues arise in a BHM when the exchangeability assumption is violated, which occurs in the presence of baskets with heterogeneous response rates. In such cases, when information is borrowed between all baskets, the type I error rate is likely to inflate as the posterior probabilities are pulled towards the common mean, μ , and away from the true treatment effect. The EXNEX model relaxes the full exchangeability assumption, allowing for some heterogeneity between treatment effects (thereby reducing type I error rate inflation) through the incorporation of the NEX component within which baskets are analysed independently, with basket-specific priors on the logit transformed response rates. Neuenschwander et al.^14^ propose setting the parameters as follows:

(2) $$m_{k}=\log\;(\frac{\rho_{k}}{1-\rho_{k}}),\;\nu_{k}^{2}=\frac{1}{\rho_{k}}+\frac{1}{1-\rho_{k}},$$

where $\rho_{k}$ is a plausible guess for the true response rate in basket k .

**Table 1.** Summary of the proposed approaches for analysis and calibration of new and existing baskets.

	Calibration		Analysis	
Approach	$\Delta_{k_{0}}$	$\Delta_{k^{\prime }}$	Existing Baskets	New Baskets
IND	EXNEX on all $k_{0}$	Independent on all $k^{\prime }$	EXNEX on all $k_{0}$	Independent on all $k^{\prime }$
UNPL	EXNEX on all $k_{0}$	$\Delta_{k_{0}}=\Delta_{k^{\prime }}$	EXNEX on all k	
PL1	EXNEX on all k		EXNEX on all k	
PL2	EXNEX on all $k_{0}$	EXNEX on all k	EXNEX on all $k_{0}$	EXNEX on all k

IND: INDependent analysis of new basket; UNPL: UNPLanned addition of new baskets; PL: PLanned addition; EXNEX: exchangeability–nonexchangeability model.

The prior probabilities, $\pi_{k}$ , for assignment to the EX/NEX component are selected prior to the trial. There is often little to no information available on the probability of exchangeability of baskets before the trial, so it is suggested to fix $\pi_{k}=0.5$ for all k baskets. Alternatively, a Dirichlet prior could be placed on these values, however, Neuenschwander et al.^19^ prove that only the mean, $\pi_{k}$ , of the distribution on the mixture weights, $\delta_{k}$ , affects inference in this case.

### 2.3. Approaches for adding a basket

We now propose four different approaches for the calibration and analysis of newly added baskets to an ongoing basket trial. In all four cases, existing baskets are analysed through an EXNEX model, however, treatment of the new basket varies. Approaches are outlined below and are summarised in Table 1.

1. **IND** - INDependent analysis of new baskets. Analyse the $K_{0}$ existing baskets by applying an EXNEX model (as in Model (1)) and calibrate $\Delta_{k_{0}}$ based on the same model. Analyse the $K^{\prime }$ new baskets independently of existing baskets (modelled as in the NEX component in Model (1)) and calibrate $\Delta_{k^{\prime }}$ based on the same model.
  Analysing the new baskets as independent may be considered desirable as it eliminates potential negative effects of smaller sample sizes in new baskets on inference in existing baskets.
2. **UNPL** – UNPLanned addition of new baskets.
  Calibrate $\Delta_{k_{0}}$ based on an EXNEX model applied to the $K_{0}$ existing baskets. When conducting analysis borrow between all K baskets through an EXNEX model. When sample sizes are equal across existing baskets, set $\Delta_{k^{\prime }}=\Delta_{k_{0}}$ for the new baskets. If sample sizes are unequal in the existing baskets, set $\Delta_{k^{\prime }}=\Delta_{i_{0}}$ where existing basket i has sample size $n_{i}$ closest to the sample size of the new basket $k^{\prime }$ , i.e. $i=\arg\;\min_{i}\{|n_{i}-n_{k^{\prime }}|\}$ .
  This is a naive analysis as $\Delta_{k_{0}}$ and $\Delta_{k\prime }$ are not adjusted to account for the additional baskets, instead these values only consider the existing baskets that began the trial. This may occur when an addition is not planned for, but once it occurs, a decision is made to borrow information from any new baskets. The motivation behind this decision would likely be linked to power requirements and the potential that borrowing carries to improve power for both new and existing baskets.
3. **PL1** - PLanned addition of new baskets where a single EXNEX model is applied.
  Calibrate $\Delta_{k_{0}}$ and $\Delta_{k^{\prime }}$ assuming that new baskets will be added during the study. To calibrate and analyse, borrow between all K baskets (new and existing) through an EXNEX model.
  The situation where it is known for certain that new baskets will be added but the timing of addition is unknown, could occur if it is apparent that a basket of patients will benefit from the study, however, are not ready in time for the commencement of the trial. This could be down to logistical issues, diagnostic techniques, or some other factors. Thus it is planned to add the basket at a later time. This approach has two subsets:
  - The time of addition of the new basket(s) is known and fixed. In this case, the sample sizes, $n_{k}$ , for each of the k=1,…,K baskets are known and fixed in the calibration procedure.
  - The time of addition of the new basket(s) is unknown. This may occur if it is desirable to add a basket as soon as it is available. In this case further simulation studies are required to explore the effect of sample size on operating characteristics, with the basket-wise type I error rate evaluated under different sample sizes. Based on these exploratory simulation studies, the trial could be calibrated under the sample size setting that resulted in the highest basket-wise type I error rate. This would ensure type I error control under all of the sample size configurations considered, but may come at the cost of reduced power if the efficacy criteria is overly conservative (i.e. too close to 1).
4. **PL2** - PLanned addition of new baskets where two EXNEX models are applied.
  Calibrate $\Delta_{k_{0}}$ based on an EXNEX model applied to the $K_{0}$ existing baskets so when analysing the existing baskets, do not borrow from any new baskets. Calibrate $\Delta_{k^{\prime }}$ based on an EXNEX model applied to all K baskets. Therefore, when analysing new baskets, information is borrowed between all baskets, new and existing. This results in two EXNEX models and, like PL1, consists of two subsets: (a) Timing of addition is known and fixed and (b) Timing of addition is unknown.
  As in IND, analysing baskets in this way will eliminate the effect on type I error rate of reduced sample sizes in the new baskets, on estimation of response rates in existing baskets. However, by allowing full information borrowing between all baskets when analysing the new baskets, one may combat the issue of lack of statistical power and precision of estimates that arises due to the limited sample size.

Both the IND and PL2 approaches utilise the same calibration and analysis models for existing baskets, with an EXNEX model applied to all $K_{0}$ existing baskets. Similarly, both the PL1 and PL2 approaches utilise the same calibration and analysis models for the new baskets, with an EXNEX model applied to all K baskets in the trial. Full model specifications are provided in the supplementary material.

### 2.4. Robust calibration procedure

A treatment is deemed effective in basket k if the posterior probability that the response rate, $p_{k}$ , is greater than $q_{0}$ , exceeds a cut-off value $\Delta_{k}$ . In a few basket trials, such as the work by Zheng and Wason^20^ and Ouma et al.,^21^ these $\Delta_{k}$ are fixed at some value, i.e. 0.975, however, an alternative is to calibrate the cut-off value in order to control some operating characteristic to a desirable level. This was implemented by Kaizer et al.,^16^ Hobbs and Landin,^17^ Chu and Yuan,^3^ Jin et al.^11^ and Berry et al.,^13^ who followed a conventional approach where $\Delta_{k}$ was calibrated under a single global null scenario in which the treatment is ineffective across all baskets. In each of these cases $\Delta_{k}$ was calibrated to achieve an 100α% type I error rate in each basket under the global null. However, this type of calibration does not guarantee type I error rate control across other scenarios when information borrowing is implemented. When borrowing information from baskets which have a heterogeneous response rate but respond positively to the treatment, the posterior probabilities are pulled upwards for baskets with an ineffective response rate compared to probabilities computed under the global null scenario, thus increasing the probability of exceeding the calibrated value, $\Delta_{k}$ . Therefore, type I error rate control is only guaranteed in the global scenario for which $\Delta_{k}$ was calibrated under, with other scenarios likely to demonstrate undesirable inflation of the type I error rate. Type I error rate inflation under the EXNEX model is observed in the simulation study conducted by Daniells et al.^12^ In this study the greatest type I error inflation is observed under a scenario in which two baskets have a response rate of 0.45, a further two have a response rate of 0.35 and just one basket is ineffective to the treatment with a response rate of 0.15. The type I error rate for the one ineffective basket was 17.3%, a substantial inflation over the nominal 10% level for which efficacy criteria were calibrated for. All non-null scenarios in this study demonstrated type I error rate inflation anywhere from 11.3-17.3% under the EXNEX model. Similar findings are presented in the simulation studies by Jin et al.^11^ and Chen and Hsiao,^22^ where efficacy criteria was again calibrated to control the type I error rate to 10% under a global null scenario. These studies presented a maximum type I error rate of 33.6% and 23.5%, respectively under the EXNEX model. Although $\Delta_{k}$ is typically calibrated to control the type I error rate, the calibration procedure remains the same for the control of any metric obtained from the posterior density such as the family-wise error rate or power.

We propose a novel calibration procedure, the RCaP, where as opposed to calibrating under a single global null scenario (which we refer to as the ‘calibration under the global null approach’), $\Delta_{k}$ is calibrated across numerous potential scenarios so that some metric, Q , is controlled *on average* across potential trial outcomes. Algorithm 1 is a guide on how to implement the RCaP, which has been generalised to account for the calibration of any metric or endpoint.

Consider a case with M simulation scenarios $p_{1},\ldots ,p_{M}$ one wishes to calibrate across. Denote the sample size and true response rate of basket k under scenario m as $n_{mk}$ and $p_{mk}$ respectively with k=1,…,K and m=1,…,M . The simulation scenarios are represented by vectors consisting of true response rate probabilities, i.e. $p_{m}=(p_{m1},\ldots ,p_{mK})$ for all m=1,…,M . The scenarios $p_{m}$ are used to generate data alongside the basket sample sizes $n_{m}=(n_{m1},\ldots ,n_{mK})$ from some data-generating function F . New data is generated using this distribution within each simulation run.

Each scenario, $p_{m}$ may carry a weight that is pre-specified by the investigators, with higher weights indicating that type I error rate control is deemed more crucial under certain scenarios. Define weights $\omega_{m}$ for each scenario m=1,…,M , where $\omega_{m}$ are positive integers. Integer values are required in Algorithm 1 for implementing RCaP, as they reflect the quantity of posterior probabilities that a scenario contributes to the calibration of efficacy criteria. A larger weight increases the contribution of a scenario relative to other scenarios in the calibration, which will provide better type I error rate control under that scenario compared to scenarios with a lower weight. If no weight is defined, set $\omega_{m}=1$ for all scenarios.

**Algorithm 1.**
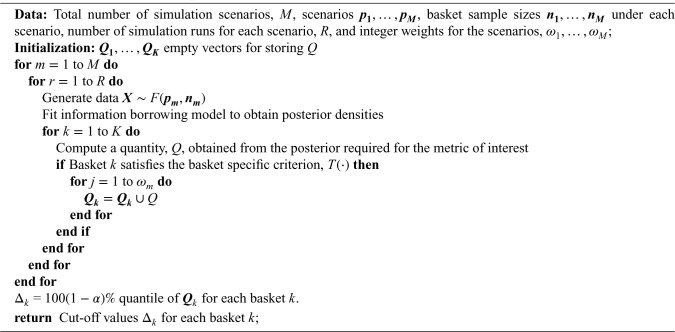
RCaP - Calibrate $\Delta_{k}$ across several simulation scenarios for any metric, Q .

Algorithm 1 requires the specification of sample sizes and the M simulation scenarios to be included, alongside their weights, $\omega_{m}$ for m=1,…,M . For a simulation scenario, $p_{m}$ , a total of R data sets are generated from $F(p_{m},n_{m})$ . A model is then fit to each of these R data sets to obtain posterior densities. Some quantity, Q , is computed from the posterior. This quantity is later used to compute the metric of interest such as the type I error rate or FWER. A binary basket-specific condition, T(⋅) is introduced which takes value one when satisfied and zero otherwise. Weights $\omega_{m}$ are utilised in the following step: If basket k satisfies T(⋅) , then $\omega_{m}$ copies of Q under each of the 1,…,K baskets are stored in vectors $Q_{1},\ldots ,Q_{K}$ . All preceding steps are repeated under each of the M simulation scenarios, thus the higher the weight $\omega_{m}$ , the more scenario m contributes to the vectors $Q_{1},\ldots ,Q_{K}$ . To compute cut-off values, $\Delta_{k}$ , the appropriate quantile is taken within each of the $Q_{k}$ vectors. As such, $\Delta_{k}$ will be the quantile of the combined quantities across all M scenarios that satisfy the basket-specific criterion (weighted by $\omega_{m}$ ), thereby controlling the metric on average across all scenarios when combined. Note that the $\Delta_{k}$ values should be set as equal for any baskets with equal sample sizes.

When the metric of interest is the type I error rate, the quantity computed is $Q=P(p_{mk}>q_{0}|X)$ . The probability of a type I error can only be computed when a basket is ineffective, thus the basket-specific condition requires that the true response rate $p_{mk}=q_{0}$ . When calibrating for type I error control, the algorithm will only use scenarios where at least one basket is ineffective to the treatment when calibrating efficacy criteria (as these are the scenarios for which the basket specific criterion is satisfied). The full algorithm applied to control the type I error rate is provided in the Supplemental material.

When utilising RCaP, one would expect superior control of the type I error rate across all scenarios compared to calibration under just the global null, as the $\Delta_{k}$ values obtained will likely be closer to 1 and hence more conservative to ensure error control across multiple scenarios rather than just the global null. With the increased conservative nature, it becomes more difficult for the posterior probability $P(p_{k}>q_{0}|X)$ to exceed $\Delta_{k}$ and deem the treatment effective. As such, a decrease in power is also likely. Both concepts are explored in the simulation studies presented in this work.

## 3. Simulation study

### 3.1. General setting

In order to explore and compare operating characteristics of the proposed approaches for handling the addition of a new basket to an ongoing trial, numerous simulation studies have been conducted. The simulation studies are split into two categories with the first category exploring the case in which the response rates in each basket are fixed to pre-defined values within the simulation study and the second category exploring the case in which the response rates are randomly generated within simulation runs. Within these simulation studies: RCaP is compared to calibration under the global null, followed by a comparison between the approaches for adding a basket to an ongoing trial. Throughout this section, all four approaches for adding baskets are considered, however, only subset (a) of PL1 and PL2 in which the time of addition is known are implemented. An exploration into the effect of timing of addition is provided in the Supplemental materials to assess the performance of PL1(b) and PL2(b).

We consider a setting with $K_{0}=4$ existing baskets and $K^{\prime }=1$ new basket added part-way through the study. Let the null and target response rates be $q_{0}=0.2$ and $q_{1}=0.4$ respectively. For existing baskets, sample sizes were fixed at $n_{k_{0}}=24$ for $k_{0}=1,\ldots ,4$ . For the new basket, $k^{\prime }=5$ , the timing of addition is known with a total of $n_{k^{\prime }}=14$ . Analysis will occur when the outcome in all 24 patients in the current baskets have been observed, as well as, all 14 patients in the new basket. These sample sizes are obtained by a Simon two-stage design^15^ with a nominal targeted type I error rate and power of 10% and 80%, respectively.

The metric considered throughout these simulation studies is the percentage of simulated data sets in which the null hypothesis is rejected ( % Reject). Further operating characteristics are presented in the Supplemental material which include: The family wise error rate, mean point estimates of the response rate in each basket and their standard deviations, as well as the percentage of simulated data sets in which the correct conclusion regarding accepting/rejecting the null was made across all K baskets ( % All Correct).

All simulations are conducted using the ‘rjags’ package v 4.13^23^ within RStudio v 1.1.453,^24^ with R v 4.1.2. Simulations consist of 10,000 simulation runs for each data scenario and approach considered.

### 3.2. Prior specification

Throughout the simulations an independent analysis model is specified such that the prior placed on the logit transformation of the response rate $p_{k}$ follows a Normal distribution: $\theta_{k}∼\text{N}(\text{logit}(0.2),10^{2})$ and is therefore centred around the null response rate with a large variance. The same prior is placed on μ in the exchangeability component of the EXNEX model with a half-normal(0,1) prior placed on σ . The prior on the NEX component is specified as in equation (2) as suggested by Neuenschwander et al.,^14^ where $\rho_{k}=0.3$ (a plausible guess for the true response rate, $p_{k}$ ) is set at a response rate considered as a marginally effective response to treatment, lying between the null and target response rate. The prior probabilities for assignment to the EX/NEX component are fixed at $\pi_{k}=0.5$ for all baskets. Full model specifications are provided in the Supplemental material.

### 3.3. Description of the fixed data scenarios simulation study

Consider a setting in which true response rates are fixed, with each basket having either a null response rate ( $p_{k}=0.2$ ) or effective response rate ( $p_{k}=0.4$ ). Scenarios 1-6 presented in Table 2, with scenarios 7–10 contributing to the calibration of efficacy criteria, as later discussed. Scenario 1 is the global null under which all baskets are ineffective, whereas, scenario 4 is the case where all baskets are truly effective. Under scenario 2, just one existing basket is truly effective, with the rest ineffective and under scenario 3 all existing baskets are effective with the new basket ineffective. Under scenario 5, all existing baskets are ineffective with the new basket effective. Finally, scenario 6 is the case where the new basket and one existing basket are effective, with the rest of the existing baskets ineffective.

**Table 2.** Simulation study scenarios: True response rates used within the simulation study.

	$p_{1}$	$p_{2}$	$p_{3}$	$p_{4}$	$p_{5}$
Scenario 1	0.2	0.2	0.2	0.2	0.2
Scenario 2	0.4	0.2	0.2	0.2	0.2
Scenario 3	0.4	0.4	0.4	0.4	0.2
Scenario 4	0.4	0.4	0.4	0.4	0.4
Scenario 5	0.2	0.2	0.2	0.2	0.4
Scenario 6	0.4	0.2	0.2	0.2	0.4
Scenario 7	0.4	0.4	0.2	0.2	0.2
Scenario 8	0.4	0.4	0.4	0.2	0.2
Scenario 9	0.4	0.4	0.2	0.2	0.4
Scenario 10	0.4	0.4	0.4	0.2	0.4

The cut-off values $\Delta_{k_{0}}$ and $\Delta_{k^{\prime }}$ are calibrated for each approach separately as described in Table 1. The calibration under the global null approach means that $\Delta_{k_{0}}$ and $\Delta_{k^{\prime }}$ are calibrated under scenario 1 to achieve 10% type I error rate. Under RCaP, an average 10% type I error rate is achieved across a number of scenarios. When implementing the RCaP procedure, consideration must be taken into which scenarios to include in the calibration. For the IND, PL1(a) and PL2(a) approaches, RCaP was implemented across scenarios 1, 2, 3, 7 and 8. As the sample size in the new basket differs from the existing baskets, these scenarios do not cover all possible partial nulls in which a basket has response rate of either 0.2 or 0.4, thus one may wish to also include scenarios in which the new basket has an effective response rate into the RCaP. This would involve including all scenarios 1–10 from Table 2 into the RCaP. A simulation study is presented in the Supplemental material that compares calibrating across scenarios 1–7 and calibrating across scenarios 1–10. Results indicated minimal differences in power and error rates and thus calibration across fewer scenarios is preferred due to the lower computational cost. Note that calibration under the UNPL approach differs from the other three approaches as its calibration only takes into account the $K_{0}=4$ existing baskets, with the new basket being an unplanned addition. Thus the four scenarios presented in Table 3 were implemented for the RCaP. These scenarios cover all global and partial nulls given K=4 baskets of equal sample size.

**Table 3.** Scenarios implemented in the RCaP for the simulation under an UNPL approach.

	$p_{1}$	$p_{2}$	$p_{3}$	$p_{4}$
Scenario 1	0.2	0.2	0.2	0.2
Scenario 2	0.4	0.2	0.2	0.2
Scenario 3	0.4	0.4	0.2	0.2
Scenario 4	0.4	0.4	0.4	0.2

RCaP: robust calibration procedure; UNPL: UNPLanned addition of new basket.

For the simulation study presented in this work, all scenarios carry the same importance and thus weights were set as $\omega_{m}=1$ for all scenarios, however, included in the Supplemental material is an exploration of these weights, demonstrating how operating characteristics changed based on their selection. To summarise, the results varied based on the approach implemented, however, placing more weight on scenarios where more baskets have an effective response rate will result in more conservative cut-off values as type I error is expected to be higher under these scenarios. In contrast, placing more weight on scenarios where the response rate is mainly ineffective across baskets, leads to less conservative cut-off values.

Although the simulation results focus on scenarios 1–6, the Supplemental material contains results for scenarios 7–10, as well as cases where a varying number of baskets have a marginally effective response to treatment.

### 3.4. Results of the fixed data scenarios simulation study

#### 3.4.1. A comparison of calibration approaches

Under the setting described above, with the six fixed response rate scenarios presented in Table 2, comparisons are now drawn between the two calibration approaches: The RCaP and calibrating under the global null. The calibration for RCaP is implemented under scenarios 1, 2, 3, 7 and 8, as described in the previous section, whilst calibration under the global null refers to calibration solely under scenario 1. The calibrated efficacy criteria for both new and existing baskets ( $\Delta_{k_{0}},\Delta_{k\prime })$ are presented in Table 4. One key observation from Table 4 is the conservative nature of the RCaP, with consistently higher efficacy criteria for all approaches. The exception to this is the efficacy criteria for the new basket under an IND approach as an independent analysis does not possess the same error inflation under non-null scenarios as the other approaches. The conservative nature of the RCaP is expected finding given that the goal is to ensure error control across not only the global null but in non-null cases too. These calibrated efficacy criteria are used across all simulation studies in this work.

**Table 4.** Calibrated $\Delta_{k_{0}}$ and $\Delta_{k^{\prime }}$ values for IND, UNPL, PL1(a) and PL2(a) under the two separate calibration methods: Calibration under the global null and the RCaP.

	Calibration under the global null		RCaP	
	$\Delta_{k_{0}}$	$\Delta_{k^{\prime }}$	$\Delta_{k_{0}}$	$\Delta_{k^{\prime }}$
IND	0.860	0.900	0.903	0.890
UNPL	0.860	0.860	0.906	0.906
PL1(a)	0.857	0.841	0.903	0.902
PL2(a)	0.860	0.841	0.903	0.902

IND: INDependent analysis of new basket; UNPL: UNPLanned addition of new baskets; PL: PLanned addition; RCaP: robust calibration procedure.

**Figure 2.**
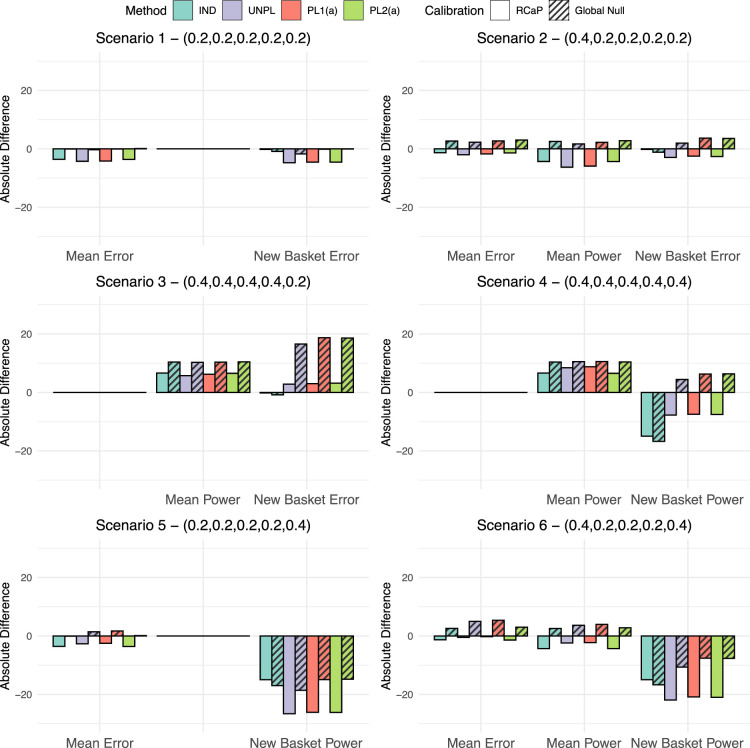
The absolute difference in type I error rate and power compared to the targeted values of 10% and 80%, respectively. This is given for all four approaches for adding a basket under the two different calibration schemes, the calibration under the global null and the robust calibration procedure (RCaP). Results are split into three categories: Mean error in which the percentage of data sets within which the null was rejected is averaged across all ineffective existing baskets; mean power as above but for all effective existing baskets and new basket error/power in which results are the percentage of data sets within which the null was rejected just in the new basket.

Given the calibrated efficacy criteria, a simulation study is now conducted, with the cut-off values under both calibration techniques implemented. For each of the six fixed scenarios presented in Table 2 and four approaches for the addition of a basket, the absolute difference between the observed type I error rate/power and the targeted level (10% and 80%, respectively) are measured under each calibration approach. These absolute differences are presented in Figure 2.

First consider the global null scenario, scenario 1. The calibration under the global null approach achieves exactly the nominal 10% type I error rate, whilst the RCaP reduces the error rate up to 4.3% of the nominal level in existing baskets and 4.7% in the new basket. Under scenario 2, RCaP results in an under-powered study, with up to a 6.3% reduction of the nominal 80% level, however, this came with a 2% decrease in type I error rate from the targeted value in existing baskets and 3.7% in the new. Whereas, calibrating under the global null inflates the error rate by up to 3% and 3.7% in existing and new baskets, respectively with a 2.7% increase in power over the nominal level.

The most blatant benefit of the RCaP is observed under scenario 3 in which the new basket is the only one with an ineffective response rate. For this basket, when calibrating under the global null, error rates are almost tripled to nearly 30% type I error rate, compared to just 13% under the RCaP. Under both calibrations, the study is over-powered, with up to a 10.4% and 6.5% increase over the nominal 80% level under the global null calibration and RCaP respectively.

In cases where the new basket is effective (scenarios 4–6), both calibration approaches lead to under-powered estimates in the new basket with the exception of scenario 4, where the power in the new baskets is increased up to 6.3% over the 80% targeted value across the IND, PL1(a) and PL2(a) approaches when calibrating under the global null. For this scenario, RCaP leads to under-powered estimates in the new basket for all four approaches. Power in existing baskets exceeds the nominal 80% value in scenario 4, with slightly higher power observed when calibrating under the global null. Under scenarios 5 and 6, RCaP reduces the type I error rate compared to the nominal level, with an absolute difference of up to a 3.6% and 1.4% reduction in scenarios 5 and 6, respectively. In scenario 6, power in existing baskets is up to a 4.3% reduction of the nominal level using the RCaP compared to an increase of 4% under a calibration under the global null approach.

Across the scenarios, estimates in existing baskets are under-powered in two cases (scenarios 2 and 6) with a maximum reduction in power of 6.3% using RCaP. Across all scenarios, power in the new basket tends to lie below the nominal 80% level under both the calibration approaches. This is due to the smaller sample size of just 14 patients. The new baskets’ power is reduced by up to 26.6% under the RCaP compared to 18.6% under the calibration under the global null. However, this comes alongside far superior control of the type I error rate across all baskets on the trial using RCaP. For existing baskets, when calibrating under the global null, the type I error rate has up to a 5.4% increase over the nominal 10% level. Whereas, RCaP controls the type I error rate at or below the nominal level across all considered scenarios for the existing baskets, whilst demonstrating a substantially lower type I error rate in the new basket across all scenarios.

The findings here corroborate previous findings in the literature in terms of the trade-off between improvements in type I error rate control and reduction in power.^10^ It is intuitive that the conservative nature of RCaP will reduce the power, however, the type I error rate control is deemed desirable compared to calibration under the global null in this work. Thus, further results presented in this work utilise the RCaP to calibrate $\Delta_{k_{0}}$ and $\Delta_{k^{\prime }}$ . Results for simulation studies in which efficacy criteria are calibrated under the global null are provided in the Supplemental material, the results demonstrate higher power but inflated type I error rate in all but the global null scenario under which the efficacy criteria was calibrated.

#### 3.4.2. A comparison of approaches for adding a basket

**Figure 3.**
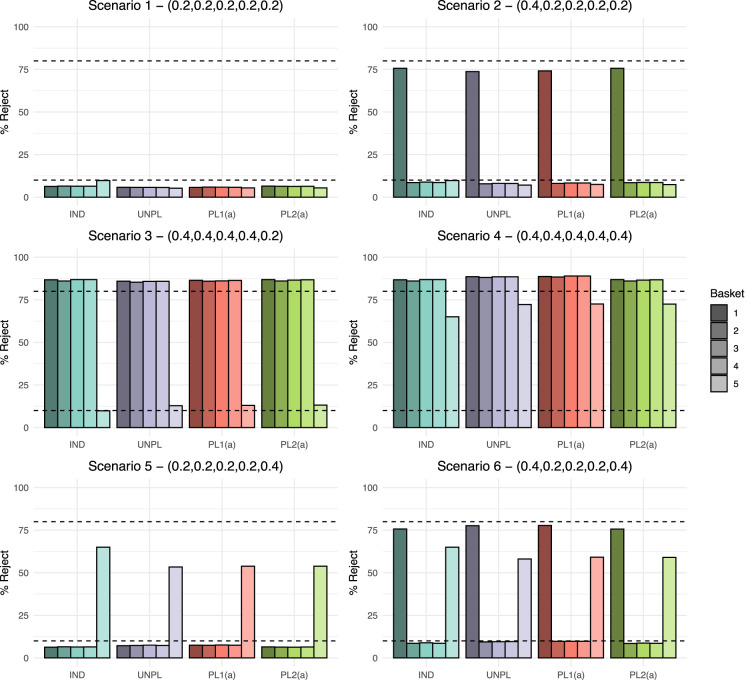
Fixed scenario simulation study results: The percentage of data sets within which the null hypothesis was rejected per basket, where $\Delta_{k_{0}}$ and $\Delta_{k^{\prime }}$ were calibrated with robust calibration procedure (RCaP) to achieve a 10% type I error rate on average. Results are provided for all four approaches for adding a basket.

We now compare the four approaches for adding a basket to an ongoing study under the six fixed data scenarios. The results for power and type I error rate for each approach are presented in Figure 3, which show the percentage of simulated data sets in which the null hypothesis was rejected. Dashed lines represent both the nominal 10% type I error rate and 80% power. Results for a further ten scenarios are presented in the Supplemental material. These additional scenarios cover different combinations of effective and ineffective new and existing baskets alongside cases in which some baskets have marginally effective response rates.

As $\Delta_{k_{0}}$ and $\Delta_{k^{\prime }}$ are calibrated using RCaP to achieve an average 10% type I error rate, in some scenarios – including the global null case – the type I error rate lies below the nominal level. However, under IND, the new basket is always analysed independently and as such, the error rate will remain at the nominal 10% level across all scenarios. Under the global null, the UNPL and PL1(a) approach in which information is borrowed between all K=5 baskets, have slightly lower type I error rates in existing baskets compared to other approaches at approximately 5.8%. UNPL, PL1(a) and PL2(a) all have similar error rates in the new basket at around 5.3%.

When analysing existing baskets, IND and PL2(a) are equivalent as they both borrow via the EXNEX model between just the four existing baskets. Under scenario 2, both approaches give the highest power at 75.7%, which does lie below the targeted 80% value, but is higher than UNPL and PL1(a) which have power of 73.7% and 74.1%, respectively. Both UNPL and PL1(a) borrow from the new basket when analysing the existing baskets. Hence, as the new basket has a null response rate, the posterior probabilities are pulled down towards the common mean resulting in lower power. Error rates for all baskets are consistent across approaches with the exception of the IND approach where the new basket type I error is approximately 3% higher as it controls type I error rate at the nominal 10% level across all scenarios.

Scenario 3 shows consistent power above the targeted 80% level in all non-null existing baskets across all four approaches. The UNPL approach demonstrates marginally lower power than other methods. The average power under UNPL is 85.7% compared to 86.2% under PL1(a). Both approaches analyse baskets in the same way, borrowing between all K baskets via the EXNEX model, the only difference being the analysis model implemented for calibration. $\Delta_{k_{0}}$ is more conservative under UNPL compared to PL1(a), leading to fewer rejections of the null hypothesis and lower power/error rates. PL1(a) and PL2(a) have marginally higher error rates in the new basket at 13.1% under scenario 3. This value is slightly lower under the UNPL approach at 12.8%, this is due to the $\Delta_{k^{\prime }}$ value being higher than PL1(a).

Under scenario 4, substantial improvement in power is observed in the new basket when information borrowing is utilised. PL1(a) gives the greatest power for all baskets. Due to the lack of information borrowing and reduced sample size in the new basket, the maximum power achieved by the IND approach is 65%. A lack of power is also evident for the new basket in scenario 5. Due the heterogeneity across new and existing baskets, the IND approach has power of 65%, which is greater than the other three approaches. Both PL1(a) and PL2(a) approaches have power of just 53.8%. Similar findings are present in scenario 6 in terms of the new basket, however both the UNPL and PL1(a) approach give slightly higher power in the existing baskets at 77.7% compared to 75.7% under an IND and PL2(a) analysis.

Overall, the largest difference in power across approaches in all scenarios is just 2%. In the presented scenarios, for existing baskets, the type I error rate is always controlled at or below the nominal level across all approaches. Differences in the type I error rate are observed in the new basket, where the IND approach always controls the type I error rate to the nominal level, whilst error inflation is present under the other three approaches in scenario 3 (type I error rate of around 13%).

### 3.5. Description of the random data scenarios simulation study

In order to further compare the performance of the four approaches for adding baskets, a second simulation study is considered. The goal of this study is to further identify where discrepancies between approaches arise. To do so, rather than fixing the true response rate for the new basket prior to the trial, it is randomly generated within each trial run of the simulation.

Following the same set-up as the fixed data scenario simulation study, four settings are considered. In each setting the response rates for existing baskets are fixed while the response rate for the new basket is randomly selected with uniform probability across an interval. Three sub-cases are considered in each setting, varying the interval from which $p_{5}$ is sampled: Sub-case (a) where the new basket is ineffective to treatment (i.e. null) so $p_{5}\in [0.1,0.2]$ , thus it is expected that the null is not rejected, sub-case (b) where the new basket has an effective response rate so $p_{5}\in [0.4,0.5]$ , thus it is expected that the null is rejected and finally sub-case (c) where the new baskets response rate lies between the null and target response rate, so $p_{5}\in [0.2,0.3]$ . The four settings are:

(1) Fix the response rate in all the existing baskets as ineffective, i.e. $p_{1,2,3,4}=0.2$ ;
(2) Fix the response rate in all the existing baskets as effective, i.e. $p_{1,2,3,4}=0.4$ ;
(3) Fix the response rate in two of the existing baskets as effective, i.e. $p_{1,2}=0.4$ and two ineffective, i.e. $p_{3,4}=0.2$ ;
(4) Fix the response rate in one of the existing baskets as effective i.e. $p_{1}=0.4$ , two as marginally effective i.e. $p_{2,3}=0.3$ and one as ineffective i.e. $p_{4}=0.2$ ,

where $p_{5}$ is varied across one of the 3 intervals (a), (b) or (c) in each of the four settings.

The efficacy criteria are obtained using RCaP, with the $\Delta_{k_{0}}$ and $\Delta_{k^{\prime }}$ from Table 4 utilised. A total of 12 simulation settings are implemented (settings 1–4 under sub-cases (a)–(c)), where in each, 10,000 random data scenarios were generated. From this, pair-wise discrepancies between approaches are identified. Pair-wise discrepancies occur when one approach concludes that the null hypothesis should be rejected in a basket, whilst another does not reject the null (hence resulting in differing efficacy conclusions). Cases where both approaches under comparison make the incorrect conclusion are not included as the aim is to identify differences between the approaches.

### 3.6. Results of the random data scenarios simulation study

**Figure 4.**
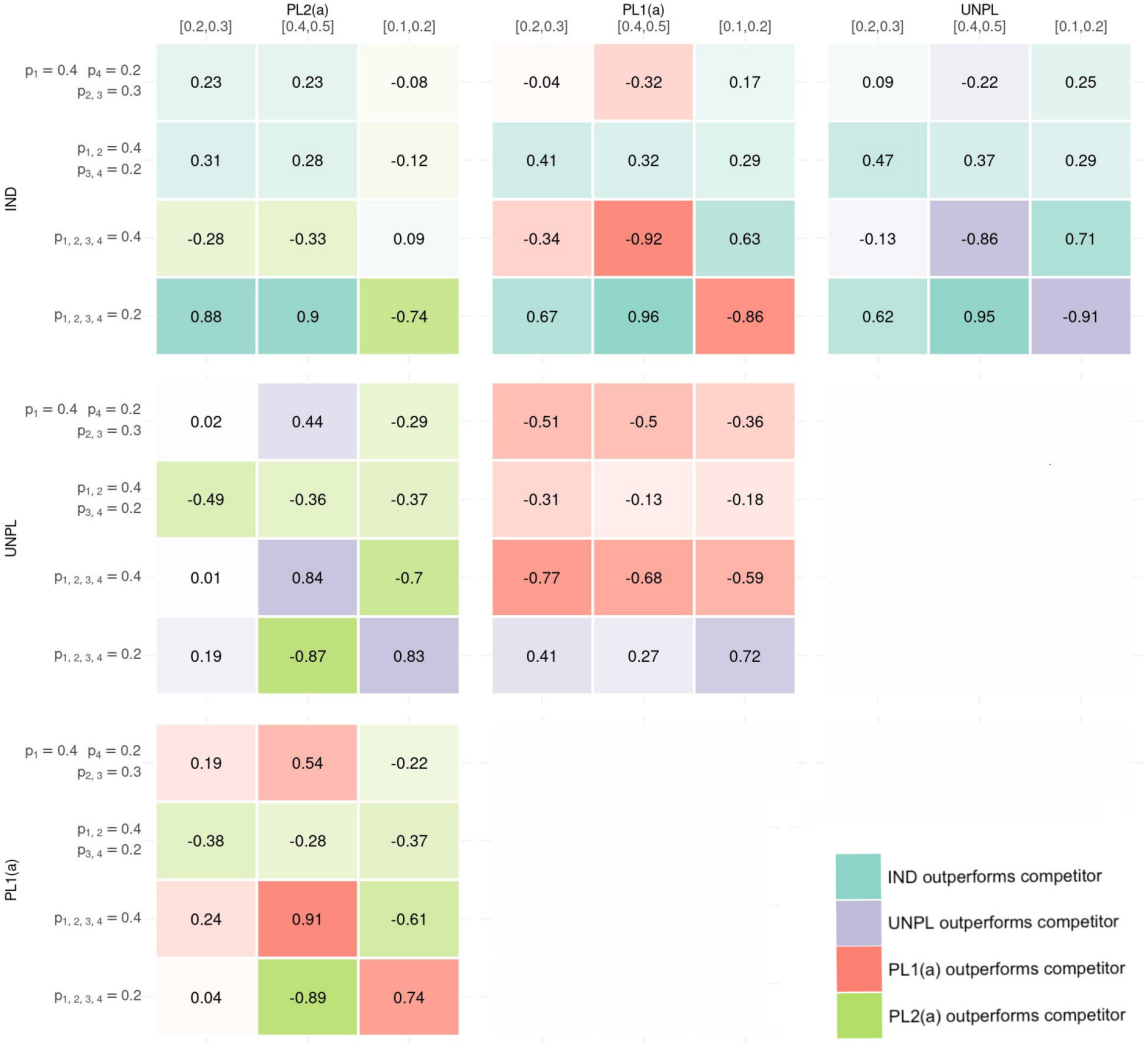
The six heat-map presents pair-wise comparisons between the four approaches for adding baskets. Within each heat-map, the results of the 12 simulation settings are presented where the metric is the difference in proportion of times the approach corresponding to rows outperformed the approach corresponding to the column (with negative values indicating the approach in the column gave more correct conclusions than the approach in the row where discrepancies between the two approaches arise). The colour in the heat map represents which approach gave superior correct conclusion, with shade representing the amount of difference between approaches. Blue represents IND giving more correct conclusions where discrepancies lie, Purple for UNPL, Red for PL1(a) and Green for PL2(b). IND: INDependent analysis of new basket; UNPL: UNPLanned addition of new baskets; PL: PLanned addition.

The pair-wise discrepancies between approaches for adding are presented as several heat maps in Figure 4. The metric of interest is the difference in proportion of correct conclusions made when discrepancies arise between the two approaches under comparison. Each sub-plot within Figure 4 represents a comparison between two approaches. Within each heat map, the colour of the cell represents the superior approach with brighter colours depicting a greater degree of difference in proportion of correct inference between the two approaches under comparison. A blue cell indicates that an IND approach is superior to its competitor approach in that setting, purple indicates that UNPL is superior, red indicates that PL1(a) is superior and green indicates that PL2(a) is superior. The values of the proportion of correct conclusions are also displayed. A negative proportion implies the approach corresponding to the column outperforms the competitor approach in the corresponding row in terms of correct conclusions made when discrepancies occurred.

Consider the pair-wise comparison between IND and UNPL. The IND approach outperforms UNPL in 8 out of the 12 simulations, making a greater proportion of correct conclusions where discrepancies occurred. In setting 1 where the existing baskets are null, the difference in approaches is substantial. For example, when the new basket is effective, IND is preferred with a difference in proportion of correct conclusion of 0.95, but when ineffective, this difference is 0.91 in favour of an UNPL approach. Other cases where UNPL is preferred over IND is when there is again homogeneity between existing and new baskets’ response rates, i.e. in setting 2 where both new and existing baskets are effective. When there is heterogeneity between all baskets, IND tends to outperform the UNPL approach.

The analysis approach in UNPL is identical to that in PL1(a), the only difference being the calibrated $\Delta_{k_{0}}$ and $\Delta_{k^{\prime }}$ values. As such, a similar pattern in results of the IND-UNPL pair-wise comparison are observed in the pair-wise comparison between IND and PL1(a). Under UNPL, the efficacy criteria is more conservative, leading to fewer rejections of the null compared to PL1(a), regardless of whether a basket is truly effective or not. The more conservative cut-off value results in the UNPL approach outperforming PL1(a) in all sub-cases of setting 1, as the ideal is for the hypothesis to not be rejected. However, in cases where at least one existing basket is effective, PL1(a) gives more correct conclusions over UNPL. This will come from the less conservative cut-off values, leading to more correct rejections.

Under the IND and PL2(a) approaches, any discrepancies that arise will come from the new basket. In settings 2–4 when at least one existing basket is effective, approaches are fairly equal in terms of difference in correct conclusions, with IND performing best when there is heterogeneity between all baskets, with the new basket effective (ranging from 0.23 to 0.31 difference in proportion of correct conclusion in favour of an PL2(a) approach). However, the Pl2(a) approach has superior performance compared to IND when all baskets have a homogeneous response.

Similarly, under PL1(a) and PL2(a), analysis for the new basket follows the same model and thus differences only lie in existing baskets. In cases of complete homogeneity between existing baskets with the new basket also having a homogeneous response rate, PL1(a) is the clear winner as power can be gained through borrowing between all baskets. However, in cases where heterogeneity is observed between response rates, such as when the new basket is effective and existing ineffective and vice-versa, PL2(a) is superior as it does not draw on information from these heterogeneous baskets when analysing existing baskets. The comparisons between UNPL and PL2(a) result in the same conclusions.

In summary, the IND approach has been identified to provide more accurate rejections of the null hypothesis when compared pair-wise to the other three approaches. In 22 out of 36 comparisons, the IND approach outperforms its competitor, with most of these cases occurring when heterogeneity is observed amongst baskets’ response rates. In cases of homogeneity amongst the response rates, the other three approaches which have stronger borrowing make more accurate rejections of the null hypothesis. In such cases PL1(a) outperforms both IND and PL2(a).

## 4. Discussion

In this work, we presented four approaches for calibration and analysis of trials when a new basket is added part-way through. Approaches utilise the EXNEX Bayesian information borrowing model which was selected for its flexible borrowing between subsets of baskets.

Through the simulation studies presented, none of the outlined approaches for adding a basket outperforms its competitors across all cases. An approach which analyses new baskets as independent whilst retaining information borrowing between existing baskets understandably has better error rate control and power in cases of heterogeneity between new and existing baskets’ response rates, with type I error rate control in the new basket guaranteed. However, significant power can be gained via information borrowing between all baskets when the new basket is homogeneous to existing ones. This is supported by results from the fixed and random data scenarios. The fixed data scenario simulation results demonstrated that when the treatment is effective for the population in the new basket, performance of the approaches vary based on the number of effective existing baskets. In our simulations, when at least half of the existing baskets were effective, higher power was observed in the new basket for the approaches that implemented information borrowing. However, when less than half of the existing baskets were effective, borrowing information reduced power by up to 7%, thus an independent approach is more appropriate. A key finding was also drawn from the random data scenario simulation study, where a planned addition of a new basket outperformed an unplanned addition in almost all settings. The exception being when all existing baskets were null. This was driven by the more conservative calibrated efficacy criteria under the UNPL approach, as both PL1(a) and UNPL follow the same analysis model. These findings are not directly comparable to the fixed data scenario simulation study as the true response rates in the new basket vary between the two studies, however, the comparison between performance remains consistent.

Throughout the simulation studies in this work, an assumption is made that the timing of addition of a new basket is known, and thus we assume a fixed sample size in each basket. In practice the calibration of efficacy criteria mostly occurs prior to the commencement of the trial, and hence before observed sample sizes are available. Due to uncertainty in the observed sample sizes, the assumption of fixed sample size has been used to conduct calibration. However, simulation studies in the Supplemental material explored the setting where timing of addition (and the sample size in the new basket) is unknown. In these simulations, the impact of sample size uncertainty is explored by monitoring the type I error rate and power as the number of patients in the new basket ranged from 1 up to the sample size of the existing baskets. It is shown that results are fairly robust to the timing of addition, with increased power in new baskets when sample sizes are larger, but consistent type I error rate and power in existing baskets regardless of the size of the new basket. This implies that the size of the new basket has no detrimental effect on baskets that opened at the start of the trial, therefore it is deduced that the main driver of error inflation in the existing baskets is heterogeneity between the new and existing baskets’ response rates rather than the sample size. As the sample size increases, the difference in error rates/power between analysing the new basket as independent and conducting information borrowing will decrease, and thus in such a case it may be beneficial to always analyse as independent to avoid issues when heterogeneity arises. In addition, should the impact of much greater or much smaller sample sizes than planned be of concern, an alternative approach could be to calibrate based on the ‘worst case scenario’ for the sample sizes (i.e. the sample size which is expected to observe the greatest type I error rate for instance).

Not considered in this work is the possibility of unequal sample sizes across existing baskets. Although unequal sample sizes would be more realistic given the setting, in our simulation studies, we opt for an equal number of patients in the existing baskets. This was chosen in order to simplify the simulation study and the number of different scenarios that would need to be considered. That being said, unequal sample sizes in basket trials with information borrowing has been explored in previous work by Daniells et al.,^12^ where it was demonstrated that a smaller basket sample size will likely result in uniformly lower power with an increased potential of type I error rate inflation as expected. We conjecture that the same findings will apply when adding new baskets. It is expected that smaller existing baskets will demonstrate more substantial improvements in power when information is borrowed from new baskets compared to baskets with an already large sample size, however, may also demonstrate greater type I error rate inflation in cases of heterogeneity amongst response rates. Should a basket be larger in size compared to others on the trial, then the benefits of borrowing information will be reduced in this basket.

Although all simulation studies conducted had just a single basket added alongside four existing baskets, a further simulation is presented in the Supplemental material, where two new baskets were added to a trial with two existing baskets. The same conclusions are drawn from the results as in the simulation studies presented in this work, but with an unplanned addition performing significantly worse than other approaches due to the lack of certainty in the calibration process with only two relatively small baskets being used. It is believed that as the ratio of existing to new baskets increases, the power gained through information borrowing in the new basket further improves due to the gain in certainty around point estimates.

We have also promoted a transition away from the traditional calibration approach in which the type I error rate is controlled under a global null scenario, towards the novel calibration technique, RCaP, where the type I error rate is controlled on average across several plausible data scenarios. The concept of calibration across several scenarios is not a wholly new concept and has been implemented extensively in the dose-finding setting, in particular when using the continual reassessment method (CRM).^25,26^ In practice, the CRM’s model parameters are calibrated to maximise the average percentage of correct doses selected across several dose-toxicity scenarios. Also, Best et al.^27^ argued for the use of average type I error rate in the pivotal study setting. They utilise average type I error rate when assessing Bayesian designs which borrow information from control or historical data. However, to the best of our knowledge the concept has not been implemented in the basket trial setting.

The proposed RCaP provides flexibility by allowing the clinician to specify potential outcomes of the trial in which one would like to control the error rate across, whilst specifying weights to these outcomes to highlight how likely they are to occur and their importance in the calibration. Throughout the simulation studies presented, equal weights across all scenarios were used. A further exploration of these weights is provided in the Supplemental material which demonstrates the important role weights play in the RCaP. To summarise the key findings, placing more weight on scenarios with fewer ineffective baskets will produce more conservative cut-off values and with that an improvement in error control but a loss in power. Putting more weight on scenarios with mostly ineffective baskets gives less conservative cut-off values and thus higher power.

The advantages of using RCaP over the calibration under the global null approach are not uniform across the scenarios or the implemented approach for adding a basket. As expected, RCaP is more advantageous over calibrating under just the global null when the true scenario differs more substantially from the global null scenario. However, the advantage of superior error control compared to the calibration under the global null approach is consistent across all scenarios, with impact on power varied based on the number of effective baskets, showing a small loss in power relative to the targeted value in a handful of cases.

Other adaptive design features, such as interim analyses with futility/efficacy stopping, are desirable and have been considered across different information borrowing methods in the basket trial setting. This includes the work by Jin et al.,^11^ Berry et al.,^13^ Chu and Yuan^3^ and Psioda et al.^28^ No such design features were included in this work, however, the methodology described here could be extended to incorporate such features. In addition, only a single treatment arm was considered in this work but the methodology can be easily extended to the multi-arm setting in which the treatment is compared to a control group. Similarly, although only a Binomial model is considered for modelling response data, more complex models such as an overdispersion model be considered. These models are useful when considering discrete data, and are used to account for unexpected variance in the responses between patients suffering from the same disease, i.e. variance across patients in the same basket.^29^ The impact of using an alternative model has not been considered, however, it is believed that the comparison between approaches of addition of a new baskets and comparison between calibration approaches will remain similar, as information borrowing can still be implemented between baskets. Finally, the only model parameters calibrated here have been the efficacy criteria, with the prior distributions and their parameters chosen as fixed. Further research into the selection of these priors/parameters could be of interest.

## Supplemental Material

sj-pdf-1-smm-10.1177_09622802251316961 - Supplemental material for How to add baskets to an ongoing basket trial with information borrowing Supplemental material, sj-pdf-1-smm-10.1177_09622802251316961 for How to add baskets to an ongoing basket trial with information borrowing by Libby Daniells, Pavel Mozgunov, Helen Barnett, Alun Bedding and Thomas Jaki in Statistical Methods in Medical Research
