# Supplementary material for: How to add baskets to an ongoing basket trial with information borrowing
Source: Stat Methods Med Res. 2025 Mar 20;34(4):717–34. doi: 10.1177/09622802251316961 (PMC12075893; doi:10.1177/09622802251316961)
Supplement: sj-pdf-1-smm-10.1177_09622802251316961 - Supplemental material for How to add baskets to an ongoing basket trial with information borrowing [file sj-pdf-1-smm-10.1177_09622802251316961.pdf]

Supplementary Material: How to Add Baskets to an Ongoing Basket Trial with Information Borrowing

Libby Daniells, Pavel Mozgunov, Helen Barnett, Alun Bedding, Thomas Jaki

Table S1: Summary of approaches for analysis and calibration when adding a basket.

Approach	Description	Calibration	Analysis
IND	Treat the new and existing baskets separately and independent of one another. Analysing the new basket as independent of existing baskets eliminates the potential negative effects of reduced information in the new baskets on existing baskets.	Calibrate Δ_{k_0} based on an EXNEX model applied to the K_0 existing baskets. For new baskets, calibrate $\Delta_{k\prime}$ based on either: (a) independent analysis conducted for each of the $K\prime$ new baskets or (b) borrow information between all $K\prime$ new baskets through a separate EXNEX model.	Analyse in the same way as calibration, with an EXNEX fitted to existing baskets and new baskets analysed with an independent model.
UNPL	Naive approach in which an unplanned addition of new baskets is made and not considered in the calibration procedure. This occurs when it is unknown a basket will be added but it is then believed that borrowing information between all baskets, new and old, can improve inference.	Calibrate Δ_{k_0} based on an EXNEX model applied to the K_0 existing baskets. Fix $\Delta_{k\prime} = \Delta_{k_0}$ once new baskets are added.	Analyse by borrowing information between all K baskets through an EXNEX model.
PL1	It is known that a basket will be added during the study and information will be borrowed between all baskets new and existing. This may occur when it is apparent that a basket of patients will benefit from the study, however is not ready in time for the commencement of the trial and thus is planned to be added at a later time.	Calibrate Δ_{k_0} and $\Delta_{k\prime}$ based on an EXNEX model applied to all K baskets. When (a) timing of addition is known: the sample sizes n_k for all K baskets are known and fixed in the EXNEX model. When (b) timing of addition is unknown: further simulation studies are required to explore the effect of $n_{k\prime}$ on operating characteristics, one could calibrate based on the least favourable configuration.	Analyse in the same way as calibration, with an EXNEX model fitted to all K baskets.
PL2	It is known that a basket will be added during the study but when conducting inference on existing baskets only information from other existing baskets is utilized, whereas for inference on new baskets, information is borrowed between all baskets in the trial. This will eliminate the effect of reduced sample sizes in new baskets on estimation of response rates in existing baskets whilst improving power and precision in the new basket.	Calibrate Δ_{k_0} based on an EXNEX model applied to just the K_0 existing baskets. Calibrate $\Delta_{k\prime}$ based on an EXNEX model applied to all K baskets. When (a) the timing of addition is known, sample sizes, n_k, for all baskets are fixed in the calibration procedure. When (b) the timing of addition is unknown, further simulation studies would be required to explore the effect of $n_{k\prime}$ on operating characteristics and adjust calibration accordingly.	Analyse in the same way as calibration with an EXNEX model fitted to just the K_0 existing baskets when analysing existing baskets and with an EXNEX model fitted to all K baskets when analysing the new basket(s).

1 Model Specification

The trial consists of a total of K baskets, divided into K_0 existing baskets and $K\prime$ new baskets. Parameter choices are those implemented throughout the simulation studies presented in the main text.

IND Calibrate and analyse based on the following model:

$$
\begin{aligned}
&Y_k \sim \text{Binomial}(n_k, p_k), && k = 1, \dots, K && \delta_{k_0} \sim \text{Bernoulli}(\pi_{k_0}),\\
&\theta_k = \log\left(\frac{p_k}{1-p_k}\right), && && M_{1k_0} \sim \text{N}(\mu, \sigma^2),\\
&\theta_{k\prime} \sim \text{N}(-1.39, 10^2), && k\prime = K_0+1, \dots, K && \mu \sim \text{N}(-1.39, 10^2),\\
&\theta_{k_0} = \delta_{k_0} M_{1k_0} + (1-\delta_{k_0}) M_{2k_0}, && k_0 = 1, \dots, K_0 && \sigma \sim \text{Half-Normal}(0,1),\\
& && && M_{2k_0} \sim \text{N}(-0.85, 4.76),
\end{aligned}
$$

with $\pi_{k_0} = 0.5$ for all $k_0 = 1, \dots, K_0$.

UNPL Calibrate based on the following model:

$$
\begin{aligned}
&Y_{k_0} \sim \text{Binomial}(n_{k_0}, p_{k_0}), && k_0 = 1, \dots, K_0 && M_{1k_0} \sim \text{N}(\mu, \sigma^2),\\
&\theta_{k_0} = \log\left(\frac{p_{k_0}}{1-p_{k_0}}\right), && && \mu \sim \text{N}(-1.39, 10^2),\\
&\theta_{k_0} = \delta_{k_0} M_{1k_0} + (1-\delta_{k_0}) M_{2k_0}, && && \sigma \sim \text{Half-Normal}(0,1),\\
&\delta_{k_0} \sim \text{Bernoulli}(\pi_{k_0}), && && M_{2k_0} \sim \text{N}(-0.85, 4.76),
\end{aligned}
$$

with $\pi_{k_0} = 0.5$ for all $k_0 = 1, \dots, K_0$. Analyse based on the following model:

$$
\begin{aligned}
&Y_k \sim \text{Binomial}(n_k, p_k), && k = 1, \dots, K && M_{1k} \sim \text{N}(\mu, \sigma^2),\\
&\theta_k = \log\left(\frac{p_k}{1-p_k}\right), && && \mu \sim \text{N}(-1.39, 10^2),\\
&\theta_k = \delta_k M_{1k} + (1-\delta_k) M_{2k}, && && \sigma \sim \text{Half-Normal}(0,1),\\
&\delta_k \sim \text{Bernoulli}(\pi_k), && && M_{2k} \sim \text{N}(-0.85, 4.76),
\end{aligned}
$$

with $\pi_k = 0.5$ for all $k = 1, \dots, K$.

PL1 Calibrate and analyse based on the following model:

$$
\begin{aligned}
&Y_k \sim \text{Binomial}(n_k, p_k), && k = 1, \dots, K && M_{1k} \sim \text{N}(\mu, \sigma^2),\\
&\theta_k = \log\left(\frac{p_k}{1-p_k}\right), && && \mu \sim \text{N}(-1.39, 10^2),\\
&\theta_k = \delta_k M_{1k} + (1-\delta_k) M_{2k}, && && \sigma \sim \text{Half-Normal}(0,1),\\
&\delta_k \sim \text{Bernoulli}(\pi_k), && && M_{2k} \sim \text{N}(-0.85, 4.76),
\end{aligned}
$$

with $\pi_k = 0.5$ for all $k = 1, \dots, K$.

PL2 Calibrate and analyse existing baskets based on the following model:

$$
\begin{aligned}
&Y_{k_0} \sim \text{Binomial}(n_{k_0}, p_{k_0}), && k_0 = 1, \dots, K_0 && M_{1k_0} \sim \text{N}(\mu, \sigma^2),\\
&\theta_{k_0} = \log\left(\frac{p_{k_0}}{1-p_{k_0}}\right), && && \mu \sim \text{N}(-1.39, 10^2),\\
&\theta_{k_0} = \delta_{k_0} M_{1k_0} + (1-\delta_{k_0}) M_{2k_0}, && && \sigma \sim \text{Half-Normal}(0,1),\\
&\delta_{k_0} \sim \text{Bernoulli}(\pi_{k_0}), && && M_{2k_0} \sim \text{N}(-0.85, 4.76),
\end{aligned}
$$

with $\pi_{k_0} = 0.5$ for all $k_0 = 1, \dots, K_0$. Calibrate and analyse new baskets based on the following model:

$$
\begin{aligned}
&Y_k \sim \text{Binomial}(n_k, p_k), \qquad k = 1, \ldots, K && M_{1k} \sim \text{N}(\mu, \sigma^2),\\
&\theta_k = \log\left(\frac{p_k}{1-p_k}\right), && \mu \sim \text{N}(-1.39, 10^2),\\
&\theta_k = \delta_k M_{1k} + (1-\delta_k) M_{2k}, && \sigma \sim \text{Half-Normal}(0,1),\\
&\delta_k \sim \text{Bernoulli}(\pi_k), && M_{2k} \sim \text{N}(-0.85, 4.76),
\end{aligned}
$$

with $\pi_k = 0.5$ for all $k = 1, \ldots, K$.

2 RCaP: Robust Calibration Procedure for Type I Error Control

Algorithm 1 describes the RCaP specifically for the control of the type I error rate.

Algorithm 1 RCaP - Calibrate Δ_k across several simulation scenarios for type I error rate control

Data: Total number of simulation scenarios, M, scenarios $\boldsymbol{p_1}, \ldots, \boldsymbol{p_M}$, basket sample sizes $\boldsymbol{n_m}$, number of simulation runs for each scenario, R, null response rate, q_0 and integer weights for the scenarios, $\omega_1, \ldots, \omega_M$;
Initialization:$\boldsymbol{Q_1}, \ldots, \boldsymbol{Q_K}$ empty vectors for storing Q
for $m = 1$ to M **do**
  for $r = 1$ to R **do**
    Generate data $\boldsymbol{X} \sim \text{Binomial}(\boldsymbol{p_m}, \boldsymbol{n_m})$
    Fit information borrowing model to obtain posterior densities
    for $k = 1$ to K **do**
      Compute the posterior probability of a type I error $\mathbb{P}(p_{mk} > q_0 | X)$, in basket k
      if $T(p_{mk} \leq q_0)$ **then**
        for $j = 1$ to ω_m **do**
          $\boldsymbol{Q_k} = \boldsymbol{Q_k} \cup \mathbb{P}(p_{mk} > q_0 | X)$
        end for
      end if
    end for
  end for
end for
Δ_k = $100(1-\alpha)\%$ quantile of $\boldsymbol{Q}_k$ for each basket k.
return Cut-off values Δ_k for each basket k;

3 Fixed Scenario Simulation Results Under the RCaP

A further 10 scenarios were considered in the fixed scenario simulation study presented in the main text. All 16 scenarios considered are presented in Table S2, where efficacy criteria were calibrated using RCaP. Tables S3 and S4 present full simulation results with all operating characteristics. Figure S1 plots the absolute difference between calibration approaches under the same scenarios and Figure S2 plots the results of the additional 10 data scenarios.

Table S2: Simulation study scenarios: Vectors of response rates used within the simulation study.

	p_1	p_2	p_3	p_4	p_5		p_1	p_2	p_3	p_4	p_5
Scenario 1	0.2	0.2	0.2	0.2	0.2	Scenario 9	0.4	0.4	0.2	0.2	0.4
Scenario 2	0.4	0.2	0.2	0.2	0.2	Scenario 10	0.4	0.4	0.4	0.2	0.4
Scenario 3	0.4	0.4	0.4	0.4	0.2	Scenario 11	0.3	0.2	0.2	0.2	0.2
Scenario 4	0.4	0.4	0.4	0.4	0.4	Scenario 12	0.3	0.3	0.2	0.2	0.2
Scenario 5	0.2	0.2	0.2	0.2	0.4	Scenario 13	0.3	0.2	0.2	0.2	0.3
Scenario 6	0.4	0.2	0.2	0.2	0.4	Scenario 14	0.3	0.3	0.2	0.2	0.3
Scenario 7	0.4	0.4	0.2	0.2	0.2	Scenario 15	0.4	0.3	0.2	0.2	0.3
Scenario 8	0.4	0.4	0.4	0.2	0.2	Scenario 16	0.4	0.3	0.3	0.2	0.3

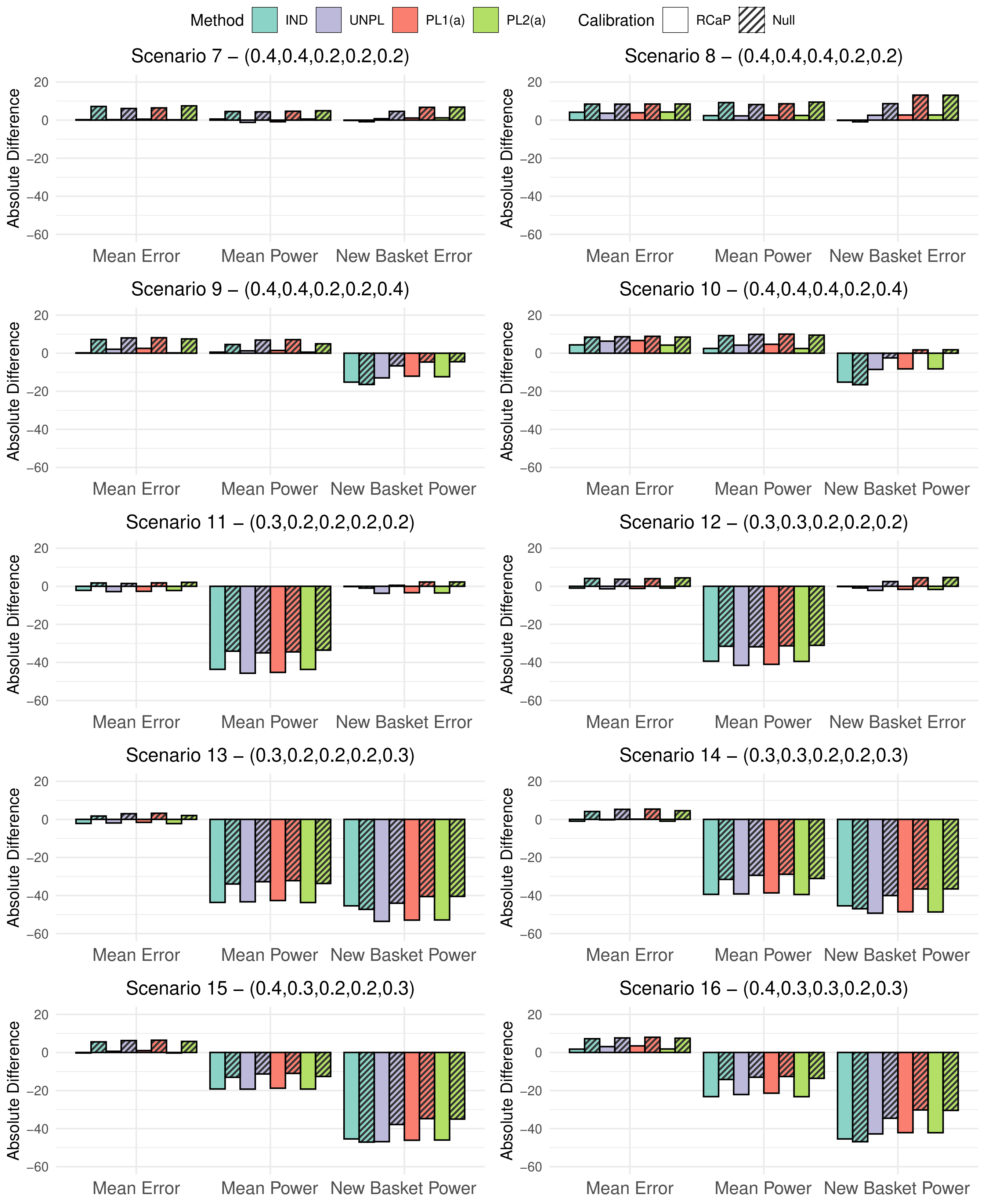

Figure S1: The absolute difference in type I error rate and power compared to the targeted values of 10% and 80% respectively. This is given for all four approaches for adding a basket under the two different calibration schemes, calibration under the global null and the RCaP. Results are split into 3 categories: mean error in which the percentage of data sets within which the null was rejected is averaged across all ineffective existing baskets; mean power as above but for all effective existing baskets and new basket error/power in which results are the percentage of data sets within which the null was rejected just in the new basket.

Comparing calibration approaches, scenarios 7 and 8 have similar findings to scenarios 1 and 2 in the main text, in terms of inflated error rates when using a calibration under the global null approach. However, error rates under the RCaP are also inflated but to a much lesser extent (e.g. 4.2% compared to 8.5% in existing baskets under PL2(a)) compared to calibration under the global null. Under scenario 9, error rates are increased by up to 8.1% of the nominal 10% level compared to a 2.5% increase under the RCaP.

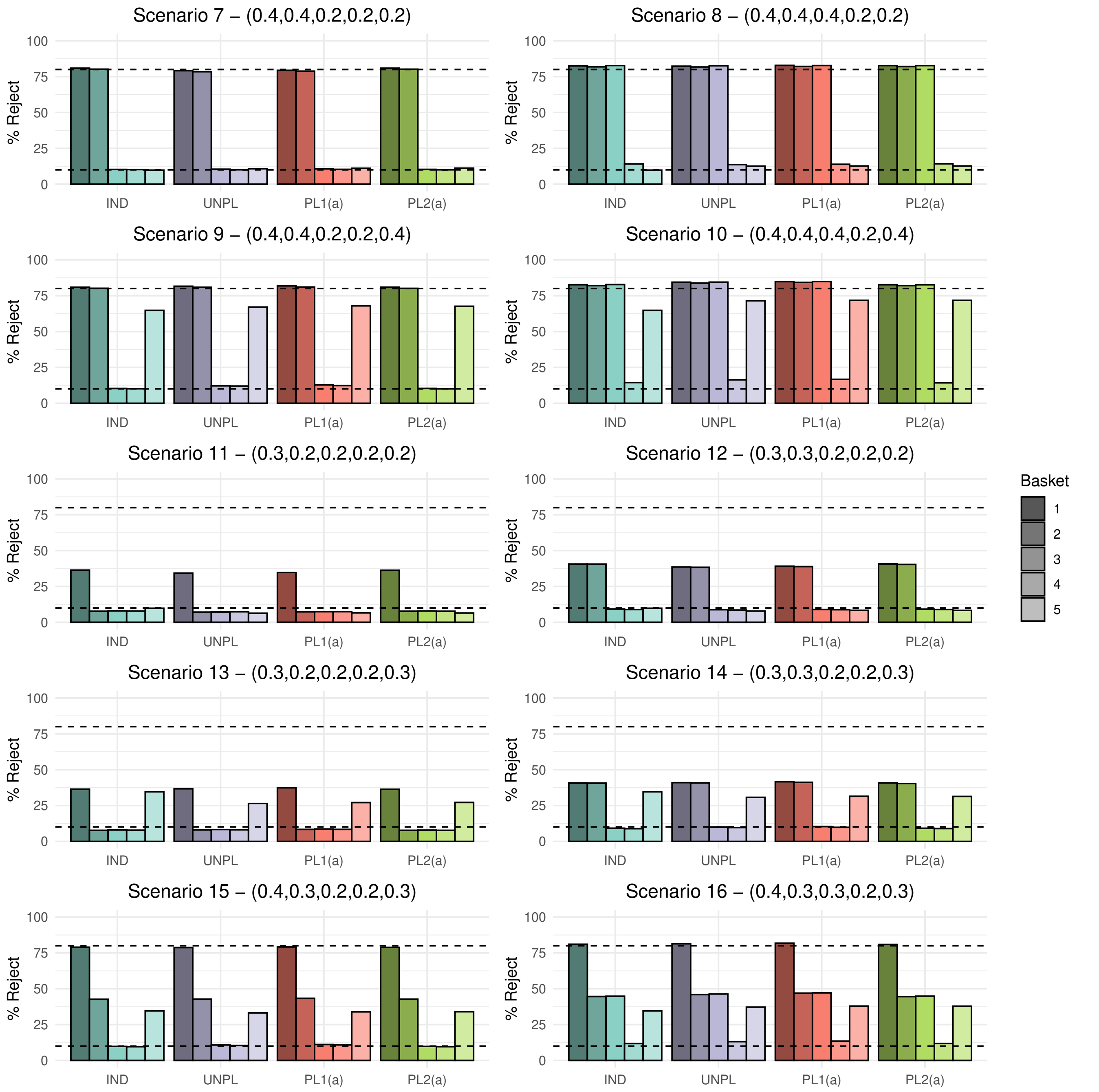

Figure S2: Fixed scenario simulation study results: The percentage of data sets within which the null hypothesis was rejected, where Δ_{k_0} and $\Delta_{k'}$ were calibrated with RCaP to achieve a 10% type I error rate on average. This is plotted for each of the four approaches for adding a basket in all five baskets.

Scenarios 11, 12, 13 and 14 are parallel to the results under scenarios 2, 7, 9 and 10 respectively. The main differences lying in the results of power, as the baskets now have a marginally effective response rate, making it more difficult to distinguish between an effective and ineffective treatment effect. In all cases, power under the RCaP is lower due to the more conservative Δ_k cut-off values, however, this came with a reduced error rate across all 4 of

these scenarios and 4 approaches. For instance, under scenario 14 error rates when calibrating under the global null have a relative increase of 5.4% over the nominal 10% level, compared to error rates controlled at or below 10% under the RCaP. Similar results are found under scenarios 14-16 in which baskets are a combination of effective, marginally effective and ineffective.

Now comparing approaches for addition of a basket, the fixed scenario results under RCaP are presented in Figure S2. Under scenario 7, error in the new basket under IND is the lowest, but the maximum inflation of the type I error rate over the 10% nominal level is only 1.1% (under PL1(a) and PL2(a), which are equivalent when analysing new baskets). Scenario 8 shows consistent power in all non-null existing baskets across all 4 approaches, all above the targeted 80% level. The UNPL approach demonstrates marginally lower power than other methods. Basket 4 has type I error rate which is slightly higher under IND and PL2(a). This is due to the common mean across baskets 1-4 being higher than across all baskets, due to the new basket being ineffective to treatment. Hence, fewer false rejections should be made under UNPL and PL1(a).

When the new basket is effective, under scenarios 9 and 10, substantial improvements in power are observed in the new basket when information borrowing is utilized. In scenarios 9 and 10, as a number of existing baskets are also effective, borrowing information between all baskets substantially improves power in the new. Under the IND and PL1(a) approaches, error rates in the existing baskets are slightly higher at around 12.5% in scenario 9 and 16.5% in scenario 10. Whereas, error rates in IND and PL2(a) have an error rate of 14.3% at the cost of reduced power in other baskets.

Results of scenarios 11, 12 and 13 correlate to those of scenarios 2, 7 and 6 respectively but with marginally effective rather than effective true response rates. This gives much lower power across baskets but similar patterns in results. Scenario 14 does differ from the results of scenario 9, with IND now producing the highest power in the new basket.

Scenarios 15 and 16 have a combination of effective, marginally effective and ineffective baskets. For existing baskets, those that are marginally effective have similar power values across all approaches under scenario 15 of around 42.7%, however, more variation is observed under scenario 16 with PL1(a) producing a power of 47%, which is higher compared to UNPL with power 46.1% and IND and PL2(a) at 44.7%. But for the single effective basket all approaches give similar power values ranging from 78.7%-79.0% under scenario 15 and 80.9%-81.8% under scenario 16. Error rates in the existing baskets are higher under the IND and PL1(a) approaches for both scenarios as the posterior probabilities are pulled up via borrowing from the effective new baskets. The maximum error rate across both scenarios is 13.5%. For the new basket UNPL, PL1 and PL2 are almost identical in power, with the IND approach giving lower power under scenario 16 due to the lack of borrowing from the mostly homogeneous existing baskets.

Table S3: Operating characteristics for the fixed scenario simulation study in the main text.

	% Reject					FWER	% Correct	Mean Point Estimate (Standard Deviation)				
**Sc 1**	**0.2**	**0.2**	**0.2**	**0.2**	**0.2**							
IND	6.33	6.52	6.42	6.46	9.82	29.37	70.63	0.202 (0.068)	0.202 (0.068)	0.202 (0.068)	0.203 (0.067)	0.200 (0.106)
UNPL	5.81	5.75	5.75	5.69	5.26	22.47	77.53	0.202 (0.065)	0.202 (0.066)	0.202 (0.065)	0.202 (0.064)	0.204 (0.079)
PL1(a)	5.73	5.92	5.89	5.78	5.45	22.82	77.18	0.202 (0.065)	0.202 (0.066)	0.202 (0.065)	0.202 (0.064)	0.204 (0.079)
PL2(a)	6.48	6.37	6.33	6.41	5.45	25.05	74.95	0.292 (0.068)	0.202 (0.068)	0.203 (0.068)	0.203 (0.067)	0.204 (0.079)
**Sc 2**	**0.4**	**0.2**	**0.2**	**0.2**	**0.2**							
IND	75.68	8.58	8.87	8.62	9.82	30.51	51.11	0.380 (0.096)	0.208 (0.072)	0.209 (0.071)	0.209 (0.070)	0.200 (0.106)
UNPL	73.73	7.83	8.12	8.01	7.07	25.47	52.69	0.376 (0.096)	0.208 (0.069)	0.209 (0.069)	0.209 (0.068)	0.212 (0.083)
PL1(a)	74.11	8.11	8.35	8.32	7.51	26.19	52.35	0.376 (0.096)	0.208 (0.069)	0.209 (0.069)	0.209 (0.068)	0.212 (0.083)
PL2(a)	75.67	8.49	8.70	8.62	7.38	27.58	52.91	0.380 (0.096)	0.208 (0.072)	0.209 (0.071)	0.209 (0.070)	0.212 (0.083)
**Sc 3**	**0.4**	**0.4**	**0.4**	**0.4**	**0.2**							
IND	86.74	86.06	86.86	86.85	9.82	9.82	54.18	0.399 (0.083)	0.398 (0.084)	0.399 (0.083)	0.399 (0.082)	0.200 (0.106)
UNPL	85.90	85.35	85.83	85.88	12.82	12.82	49.08	0.394 (0.082)	0.393 (0.083)	0.394 (0.082)	0.394 (0.081)	0.241 (0.096)
PL1(a)	86.45	85.92	86.12	86.42	13.00	13.00	50.33	0.394 (0.082)	0.393 (0.083)	0.394 (0.082)	0.394 (0.081)	0.241 (0.096)
PL2(a)	86.84	86.02	86.56	86.73	13.17	13.17	51.89	0.399 (0.083)	0.398 (0.084)	0.399 (0.083)	0.399 (0.082)	0.241 (0.096)
**Sc 4**	**0.4**	**0.4**	**0.4**	**0.4**	**0.4**							
IND	86.74	86.06	86.86	86.85	65.03	39.58		0.399 (0.083)	0.398 (0.084)	0.399 (0.083)	0.399 (0.082)	0.400 (0.131)
UNPL	88.57	88.12	88.53	88.51	72.25	47.45		0.399 (0.080)	0.399 (0.080)	0.399 (0.080)	0.400 (0.078)	0.398 (0.098)
PL1(a)	88.71	88.41	88.97	88.99	72.52	48.03		0.399 (0.080)	0.398 (0.080)	0.399 (0.079)	0.399 (0.078)	0.398 (0.098)
PL2(a)	86.84	86.02	86.56	86.73	72.46	44.33		0.399 (0.083)	0.398 (0.084)	0.399 (0.083)	0.399 (0.082)	0.398 (0.098)
**Sc 5**	**0.2**	**0.2**	**0.2**	**0.2**	**0.4**							
IND	6.33	6.52	6.42	6.46	65.03	21.49	50.86	0.202 (0.068)	0.202 (0.068)	0.202 (0.068)	0.203 (0.067)	0.400 (0.131)
UNPL	7.16	7.28	7.51	7.31	53.41	24.19	38.22	0.207 (0.067)	0.206 (0.067)	0.207 (0.067)	0.207 (0.066)	0.365 (0.119)
PL1(a)	7.48	7.42	7.59	7.47	53.88	24.67	38.13	0.207 (0.067)	0.206 (0.067)	0.207 (0.067)	0.207 (0.066)	0.365 (0.119)
PL2(a)	6.48	6.37	6.33	6.41	53.84	21.29	40.94	0.202 (0.068)	0.202 (0.068)	0.203 (0.068)	0.203 (0.067)	0.365 (0.119)
**Sc 6**	**0.4**	**0.2**	**0.2**	**0.2**	**0.4**							
IND	75.68	8.58	8.87	8.62	65.03	22.89	36.84	0.380 (0.096)	0.208 (0.072)	0.209 (0.071)	0.209 (0.070)	0.400 (0.131)
UNPL	77.61	9.43	9.53	9.61	58.07	23.97	32.17	0.379 (0.093)	0.213 (0.071)	0.214 (0.071)	0.214 (0.070)	0.372 (0.115)
PL1(a)	77.73	9.75	9.75	9.75	59.16	24.28	33.31	0.379 (0.093)	0.213 (0.071)	0.214 (0.071)	0.214 (0.070)	0.372 (0.115)
PL2(a)	75.67	8.49	8.70	8.62	59.00	22.81	32.46	0.380 (0.096)	0.208 (0.072)	0.209 (0.071)	0.209 (0.070)	0.372 (0.115)
**Sc 7**	**0.4**	**0.4**	**0.2**	**0.2**	**0.2**							
IND	80.95	80.17	10.31	10.18	9.82	26.15	37.97	0.386 (0.092)	0.385 (0.093)	0.216 (0.075)	0.216 (0.074)	0.200 (0.106)
UNPL	79.17	78.44	10.46	10.06	10.74	26.14	44.87	0.381 (0.092)	0.380 (0.092)	0.216 (0.072)	0.216 (0.072)	0.221 (0.087)
PL1(a)	79.44	78.85	10.66	10.36	11.10	26.82	44.77	0.381 (0.092)	0.380 (0.092)	0.216 (0.072)	0.216 (0.072)	0.221 (0.087)
PL2(a)	80.95	80.15	10.34	10.04	11.18	27.06	46.64	0.386 (0.092)	0.385 (0.093)	0.216 (0.075)	0.216 (0.074)	0.221 (0.087)
**Sc 8**	**0.4**	**0.4**	**0.4**	**0.2**	**0.2**							
IND	82.50	81.89	82.74	14.15	9.82	22.64	41.66	0.392 (0.088)	0.391 (0.089)	0.392 (0.087)	0.223 (0.078)	0.200 (0.106)
UNPL	82.34	81.77	82.57	13.62	12.57	23.99	40.52	0.387 (0.087)	0.386 (0.088)	0.387 (0.087)	0.224 (0.076)	0.231 (0.092)
PL1(a)	82.86	82.13	82.78	13.88	12.68	24.32	40.75	0.387 (0.087)	0.386 (0.088)	0.387 (0.087)	0.224 (0.076)	0.231 (0.092)
PL2(a)	82.68	82.04	82.66	14.24	12.69	25.18	40.15	0.392 (0.088)	0.391 (0.088)	0.392 (0.087)	0.224 (0.078)	0.231 (0.092)
**Sc 9**	**0.4**	**0.4**	**0.2**	**0.2**	**0.4**							
IND	80.91	80.19	10.3	10.12	64.80	18.17	34.09	0.385 (0.092)	0.385 (0.093)	0.216 (0.075)	0.216 (0.074)	0.399 (0.131)
UNPL	81.61	80.89	12.17	12.00	67.04	21.26	34.35	0.385 (0.089)	0.384 (0.089)	0.222 (0.075)	0.221 (0.074)	0.381 (0.109)
PL1(a)	81.86	81.04	12.78	12.3	67.93	22.05	34.41	0.385 (0.089)	0.384 (0.089)	0.222 (0.075)	0.222 (0.074)	0.381 (0.109)
PL2(a)	80.95	80.15	10.34	10.04	67.65	18.14	36.79	0.386 (0.092)	0.385 (0.093)	0.216 (0.075)	0.216 (0.074)	0.381 (0.109)

Table S4: Operating characteristics for the fixed scenario simulation study in the main text.

	% Reject					FWER	% Correct	Mean Point Estimate (Standard Deviation)				
**Sc 10**	**0.4**	**0.4**	**0.4**	**0.2**	**0.4**							
IND	82.68	82.04	82.78	14.37	64.80	14.37	30.36	0.392 (0.088)	0.391 (0.089)	0.392 (0.087)	0.223 (0.078)	0.399 (0.131)
UNPL	84.43	83.79	84.46	16.33	71.47	16.33	36.58	0.393 (0.084)	0.392 (0.085)	0.392 (0.084)	0.228 (0.078)	0.390 (0.104)
PL1(a)	84.83	84.30	84.90	16.67	71.77	16.67	37.28	0.393 (0.084)	0.392 (0.085)	0.392 (0.084)	0.228 (0.078)	0.390 (0.104)
PL2(a)	82.68	82.04	82.66	14.24	71.77	14.24	33.97	0.392 (0.088)	0.391 (0.088)	0.392 (0.087)	0.224 (0.078)	0.390 (0.104)
**Sc 11**	**0.3**	**0.2**	**0.2**	**0.2**	**0.2**							
IND	36.38	7.73	7.98	7.84	9.83	28.40	23.71	0.287 (0.083)	0.206 (0.069)	0.207 (0.069)	0.207 (0.068)	0.200 (0.106)
UNPL	34.33	7.12	7.19	7.33	6.32	22.86	23.15	0.284 (0.081)	0.206 (0.067)	0.207 (0.066)	0.207 (0.065)	0.210 (0.080)
PL1(a)	34.77	7.29	7.38	7.39	6.63	23.42	23.22	0.284 (0.081)	0.206 (0.067)	0.207 (0.066)	0.207 (0.065)	0.210 (0.080)
PL2(a)	36.32	7.74	7.88	7.73	6.51	24.98	24.33	0.287 (0.083)	0.206 (0.069)	0.207 (0.069)	0.207 (0.068)	0.210 (0.080)
**Sc 12**	**0.3**	**0.3**	**0.2**	**0.2**	**0.2**							
IND	40.65	40.61	9.20	8.87	9.83	24.55	13.63	0.291 (0.081)	0.291 (0.082)	0.212 (0.070)	0.212 (0.069)	0.200 (0.106)
UNPL	38.59	38.36	8.78	8.56	7.85	21.50	12.14	0.288 (0.079)	0.287 (0.080)	0.212 (0.067)	0.212 (0.067)	0.215 (0.082)
PL1(a)	39.15	38.87	8.95	8.78	8.38	22.16	12.23	0.288 (0.079)	0.287 (0.080)	0.212 (0.067)	0.212 (0.067)	0.215 (0.081)
PL2(a)	40.72	40.36	9.16	8.94	8.34	22.83	13.34	0.291 (0.081)	0.290 (0.082)	0.212 (0.070)	0.212 (0.069)	0.215 (0.081)
**Sc 13**	**0.3**	**0.2**	**0.2**	**0.2**	**0.3**							
IND	36.38	7.73	7.98	7.84	34.58	20.65	8.66	0.287 (0.083)	0.206 (0.069)	0.207 (0.069)	0.207 (0.068)	0.300 (0.122)
UNPL	36.70	8.00	8.26	8.05	26.43	20.68	7.47	0.287 (0.080)	0.209 (0.067)	0.210 (0.067)	0.210 (0.066)	0.285 (0.097)
PL1(a)	37.36	8.37	8.53	8.38	27.08	21.40	7.77	0.287 (0.080)	0.209 (0.067)	0.210 (0.067)	0.210 (0.066)	0.285 (0.097)
PL2(a)	36.32	7.74	7.88	7.73	27.16	20.54	7.18	0.287 (0.083)	0.206 (0.069)	0.207 (0.069)	0.207 (0.068)	0.285 (0.097)
**Sc 14**	**0.3**	**0.3**	**0.2**	**0.2**	**0.3**							
IND	40.65	40.61	9.20	8.87	34.58	16.34	5.08	0.291 (0.081)	0.291 (0.082)	0.212 (0.070)	0.212 (0.069)	0.300 (0.122)
UNPL	40.97	40.71	9.93	9.58	30.72	17.53	5.32	0.291 (0.078)	0.290 (0.079)	0.215 (0.068)	0.215 (0.067)	0.290 (0.095)
PL1(a)	41.60	41.16	10.33	9.82	31.45	17.96	5.62	0.291 (0.078)	0.290 (0.079)	0.215 (0.068)	0.215 (0.067)	0.290 (0.095)
PL2(a)	40.72	40.36	9.16	8.94	31.36	16.37	5.79	0.291 (0.081)	0.290 (0.082)	0.212 (0.070)	0.212 (0.069)	0.290 (0.095)
**Sc 15**	**0.4**	**0.3**	**0.2**	**0.2**	**0.3**							
IND	78.98	42.68	9.82	9.60	34.58	17.44	9.32	0.382 (0.093)	0.295 (0.083)	0.214 (0.072)	0.214 (0.072)	0.300 (0.122)
UNPL	78.74	42.73	10.77	10.45	33.14	18.92	9.94	0.378 (0.091)	0.295 (0.080)	0.217 (0.071)	0.217 (0.070)	0.295 (0.096)
PL1(a)	79.17	43.29	11.11	10.86	33.89	19.54	10.03	0.378 (0.091)	0.295 (0.080)	0.217 (0.071)	0.217 (0.070)	0.295 (0.096)
PL2(a)	78.84	42.72	9.79	9.61	34.00	17.46	10.98	0.382 (0.093)	0.295 (0.083)	0.214 (0.072)	0.214 (0.071)	0.295 (0.096)
**Sc 16**	**0.4**	**0.3**	**0.3**	**0.2**	**0.3**							
IND	81.03	44.59	44.79	11.75	34.58	11.75	4.65	0.383 (0.090)	0.299 (0.081)	0.300 (0.080)	0.219 (0.073)	0.300 (0.122)
UNPL	81.36	45.96	46.39	13.08	37.22	13.08	7.89	0.390 (0.088)	0.299 (0.078)	0.300 (0.077)	0.222 (0.071)	0.301 (0.094)
PL1(a)	81.78	46.94	47.1	13.46	37.88	13.46	8.55	0.380 (0.088)	0.300 (0.078)	0.300 (0.077)	0.222 (0.071)	0.301 (0.094)
PL2(a)	80.93	44.52	44.87	11.83	37.84	11.83	5.69	0.383 (0.091)	0.299 (0.081)	0.300 (0.080)	0.219 (0.073)	0.301 (0.094)

4 Comparison of Using Differing Number of Scenarios in the RCaP

Simulation studies in the main text were conducted under the novel robust calibration procedure (RCaP) in order to achieve a 10% type I error rate on average across several scenarios. RCaP was implemented under scenarios 1, 2, 3, 7 and 8 in the main text. These scenarios included all global and partial nulls assuming equal sample sizes across baskets. However, due to the new basket having a reduced sample size, these scenarios no longer cover all partial and global nulls. This is resolved by also including scenarios 5, 6, 9 and 10 in the calibration procedure. Exploration is now conducted into differences in performance based on the number of scenarios incorporated into RCaP.

Note that under UNPL, calibration differs as it consists of just the four existing baskets. The equal sample size across baskets, results in just 4 global and partial null scenarios and thus Δ_{k_0} is calibrated just across these four scenarios. Results presented incorporate the irrelevant difference between calibration in UNPL, with absolute difference values given as 0 throughout.

For all approaches, $\Delta_{k'}$ values are equal under both calibrations. Under scenarios 5, 6, 9 and 10, the baskets response rate is effective and thus not included when taking the quantile to obtain $\Delta_{k\prime}$, therefore, only including scenarios 1, 2, 3, 7 and 8 in the calibration.

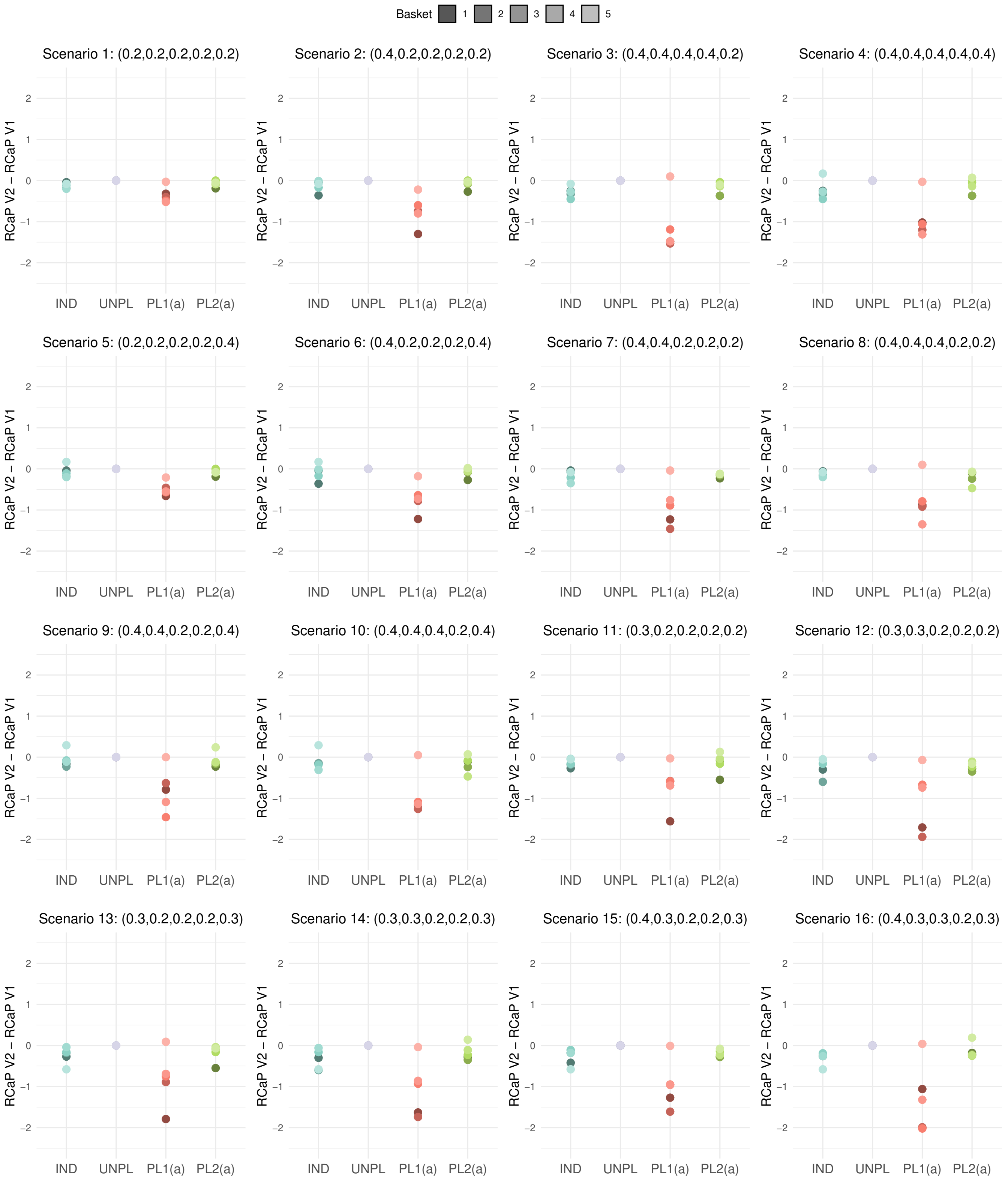

Figure S3: Absolute difference in the number of simulated data sets within which the null hypothesis is rejected between an RCaP under scenarios 1, 2, 3, 7 and 8 (RCaP V1) and an RCaP under scenarios 1-10 (RCaP V2), excluding the global alternative. This is split by approach and basket.

Figure S3 presents the absolute difference in percentage rejections of the null under an RCaP under scenarios 1, 2, 3, 7 and 8 and an RCaP under scenarios 1-10 (excluding the global alternative). In all bar a handful of cases, the

percentage rejections i.e. type I error rate and power are lower under an RCaP under 1-10 vs the RCaP with fewer scenarios.

Table S5: Calibrated Δ_{k_0} and $\Delta_{k'}$ values for each of the approaches for adding a basket under an RCaP under scenarios 1, 2, 3, 7 and 8 and an RCaP under scenarios 1-10

.

	RCaP across 1-10		RCaP across 1,2,3,7,8	
	Δ_{k_0}	$\Delta_{k'}$	Δ_{k_0}	$\Delta_{k'}$
IND	0.9044	0.8989	0.9030	0.8989
UNPL	0.9056	0.9056	0.9056	0.9056
PL1(a)	0.9101	0.9021	0.9034	0.9021
PL2(a)	0.9044	0.9021	0.9030	0.9021

Differences under IND and PL2 are always less than 1%, a negligible difference. This is expected due to the very similar Δ_{k_0} and $\Delta_{k'}$ values obtained under both calibration cases (see Table S5). However, more differences are observed under PL1(a), reaching up to 2% (scenario 16 in a marginally effective basket). This is due to the more conservative cut-off value. So even in the worst cases, differences between approaches are rather small. Calibration across fewer scenarios is less computationally expensive as it considers four fewer data scenarios compared to a calibration across scenarios 1-10 (excluding scenario 4, the global alternative). Due to the very minute differences between approaches, particularly in IND and PL2(a), a calibration across scenarios 1, 2, 3, 7 and 8 is recommended for it's reduced computational time.

5 Simulation Results Using Different Scenario Weights in the RCaP

In all simulation studies presented in the main text, for the RCaP equal weights, ω_i, for each of the i scenarios are implemented in the procedure. Equal weights implies each of the scenarios carries the same importance in the calibration, this may be the case if each scenario is equally likely to occur in the trial. This section explores the effect of altering these weights on the performance of the calibration procedure.

The simulation studies presented all implemented RCaP across the 5 scenarios presented in Table S6, with equal weights $\omega_1 = \omega_2 = \omega_3 = \omega_4 = \omega_5 = 1$ implemented. These weights are now varied to put more importance on certain

Table S6: Simulation study scenarios included in the RCaP in the main text.

	p_1	p_2	p_3	p_4	p_5
Scenario 1	0.2	0.2	0.2	0.2	0.2
Scenario 2	0.4	0.2	0.2	0.2	0.2
Scenario 3	0.4	0.4	0.2	0.2	0.2
Scenario 4	0.4	0.4	0.4	0.2	0.2
Scenario 5	0.4	0.4	0.4	0.4	0.2

scenarios relative to others. Tables S7, S8, S9 and S10 summarise the operating characteristics of each of the four approaches for adding: IND, UNPL, PL1 and PL2, under different weight settings. Presented are the calibrated cut-off values obtained and the mean type I error rate and power (split by new and existing baskets). Note that the mean is taken across scenarios 1-10 presented in Table S2 in which the basket has either an effective or ineffective response rate.

Consider first the IND approach for adding a basket. As displayed in Table S7, cut-off values, mean error and mean power are identical for the new basket across all weight combinations. Under the IND approach, new baskets are analysed as independent, which guarantees error control to the nominal level in the new basket in all scenarios in which the true response rate is q_0. As such, the cut-off value obtained under each of the 5 scenarios under considered will be equal, so altering the weight will have no impact. However, operating characteristics in the existing baskets are affected by the weight choice. As mentioned in the main text, the type I error rate increases with the number of effective existing baskets, thus scenarios 3 and 4 will display greater error rates than say scenarios 1 and 2. Placing more weight on the scenarios with only 2 or 3 ineffective baskets (i.e. where error inflation is expected to be the greatest) results in a more conservative cut-off value, Δ_{k_0}, in order to ensure error control. With this a reduction in power is observed compared to equal weights. Under equal weights, the mean power is 83.2%, whereas placing double the weight on scenario 4 results in a power of 82.1%. Placing 4 times the weight on this scenario decreases the power further to 81.2%. If more weight is placed on scenario 1 (where the type I error rate is expected to be lowest due to all baskets being null), Δ_{k_0} is less conservative than equal weights, resulting in a higher mean error of 9.4% compared to 8.8% but with an increase in power of 84.2%.

Table S7: IND: Summary of operating characteristics under several weight combinations ($\boldsymbol{\omega} = (\omega_1, \omega_2, \omega_3, \omega_4, \omega_5)$) for the 5 scenarios included in the RCaP in the main text.

IND			Mean Error		Mean Power	
$\boldsymbol{\omega}$	Δ_{k_0}	$\Delta_{k'}$	Existing	New	Existing	New
(1,1,1,1,1)	0.902	0.899	8.75	9.97	83.18	65.19
(2,1,1,1,1)	0.896	0.899	9.39	9.97	84.15	65.19
(1,2,1,1,1)	0.900	0.899	8.99	9.97	83.54	65.19
(1,1,2,1,1)	0.902	0.899	8.73	9.97	83.15	65.19
(1,1,1,2,1)	0.908	0.899	8.12	9.97	82.12	65.19
(1,1,1,1,2)	0.902	0.899	8.75	9.97	83.18	65.19
(1,1,1,1,4)	0.902	0.899	8.75	9.97	83.18	65.19
(1,1,1,4,1)	0.913	0.899	7.64	9.97	81.20	65.19
(1,1,1,2,2)	0.908	0.899	8.12	9.97	82.12	65.19

Under an unplanned addition, the calibrated cut-off values for the UNPL approach do vary based on the weights implemented in RCaP for all baskets existing and new. Like in the IND approach, placing more weights on scenarios 1 and 2 gives less conservative cut-off values for all baskets resulting in higher error with higher power compared to equal

weights. Similarly, placing more weight on scenarios with fewer ineffective baskets requires more conservative cut-off values to ensure error control with a lower power also observed. As cut-off values are calibrated based on just the existing baskets, any scenarios which put equal weight on existing baskets will be equivalent to the $\boldsymbol{\omega} = (1, 1, 1, 1, 1)$ case, regardless of the choice of ω_5.

Table S8: UNPL: Summary of operating characteristics under several weight combinations ($\boldsymbol{\omega} = (\omega_1, \omega_2, \omega_3, \omega_4, \omega_5)$) for the 5 scenarios included in the RCaP in the main text.

UNPL			Mean Error		Mean Power	
$\boldsymbol{\omega}$	Δ_{k_0}	$\Delta_{k'}$	Existing	New	Existing	New
(1,1,1,1,1)	0.900	0.900	9.63	10.09	84.33	65.38
(2,1,1,1,1)	0.893	0.893	10.40	10.88	85.30	66.62
(1,2,1,1,1)	0.897	0.897	9.89	10.34	84.69	65.80
(1,1,2,1,1)	0.901	0.901	9.47	9.99	84.13	65.16
(1,1,1,2,1)	0.907	0.907	8.87	9.59	83.23	64.32
(1,1,1,1,2)	0.900	0.900	9.63	10.09	84.33	65.38
(1,1,1,1,4)	0.900	0.900	9.63	10.09	84.33	65.38
(1,1,1,4,1)	0.912	0.912	8.23	9.22	82.20	63.40
(1,1,1,2,2)	0.907	0.907	8.87	9.59	83.23	64.32

PL1(a) borrows information between all baskets therefore, changing the weights in all scenarios will result in differing operating characteristics. Similar findings in terms of conservative calibrated cut-offs to the IND and UNPL approach are drawn. When ω_5 is increased relative to the weights on other scenarios, the cut-off value is again more conservative than an equal weight scenario, particularly for the new basket. In scenarios 5, only the new basket is ineffective, thus this scenario only contributes to the calibration of $\Delta_{k'}$ and does not impact Δ_{k_0}. Under $\boldsymbol{\omega} = (1, 1, 1, 1, 2)$, $\Delta_{k'}$ increases to 0.909 compared to 0.901 under equal weights and further increases to 0.923 under $\boldsymbol{\omega} = (1, 1, 1, 1, 4)$, resulting in a lower power in the new basket of 61.2% compared to 65.17% under equal weights. The most conservative Δ_{k_0} is observed under $\boldsymbol{\omega} = (1, 1, 1, 4, 1)$ in which $\Delta_{k_0} = 0.912$ resulting in 82.2% power in existing baskets compared to 84.2% under equal weights. Identical conclusions are drawn for the PL2(a) approach as displayed in Table S10.

Table S9: PL1(a): Summary of operating characteristics under several weight combinations ($\boldsymbol{\omega} = (\omega_1, \omega_2, \omega_3, \omega_4, \omega_5)$) for the 5 scenarios included in the RCaP in the main text.

PL1(a)			Mean Error		Mean Power	
$\boldsymbol{\omega}$	Δ_{k_0}	$\Delta_{k'}$	Existing	New	Existing	New
(1,1,1,1,1)	0.900	0.901	9.57	9.99	84.22	65.17
(2,1,1,1,1)	0.894	0.894	10.37	10.83	85.24	66.48
(1,2,1,1,1)	0.898	0.897	9.87	10.42	84.62	65.84
(1,1,2,1,1)	0.901	0.903	9.46	9.80	84.08	64.82
(1,1,1,2,1)	0.907	0.908	8.51	9.50	83.19	64.08
(1,1,1,1,2)	0.901	0.909	9.57	9.45	84.22	63.95
(1,1,1,1,4)	0.901	0.923	9.57	8.41	84.22	61.20
(1,1,1,4,1)	0.912	0.919	8.19	8.76	82.20	62.09
(1,1,1,2,2)	0.907	0.916	8.85	9.00	83.29	62.73

To summarise, weights do play an important role in the RCaP procedure and can be utilized in order to influence error control and power improvement. As seen, placing more weight on scenarios with fewer ineffective baskets will improve error control with a cost of reduced power, whilst putting more weight on scenarios with mostly ineffective baskets gives better power. Should information be available regarding which scenarios are most likely to occur, these weights could be specified in order to improve trial inference.

Table S10: PL2(a): Summary of operating characteristics under several weight combinations ($\boldsymbol{\omega} = (\omega_1, \omega_2, \omega_3, \omega_4, \omega_5)$) for the 5 scenarios included in the RCaP in the main text.

PL2(a)			Mean Error		Mean Power	
$\boldsymbol{\omega}$	Δ_{k_0}	$\Delta_{k'}$	Existing	New	Existing	New
(1,1,1,1,1)	0.902	0.901	8.71	10.00	83.14	65.17
(2,1,1,1,1)	0.896	0.893	9.36	10.83	84.11	66.60
(1,2,1,1,1)	0.900	0.897	8.96	10.41	83.54	65.82
(1,1,2,1,1)	0.903	0.903	8.67	9.81	83.09	64.88
(1,1,1,2,1)	0.908	0.908	8.15	9.49	82.14	64.02
(1,1,1,1,2)	0.902	0.909	8.71	9.44	83.14	63.85
(1,1,1,1,4)	0.902	0.924	8.71	8.40	83.14	62.74
(1,1,1,4,1)	0.913	0.919	7.63	8.76	81.22	62.08
(1,1,1,2,2)	0.908	0.916	8.15	9.00	82.14	62.74

6 Fixed Scenario Simulation Results Under a Calibration Under the Global Null Approach

Results presented in the main text utilized the RCaP in which the type I error rate is controlled on average across several data scenarios. The results under a calibration under the global null approach in which type I error rate is controlled under a global null scenario, are presented here.

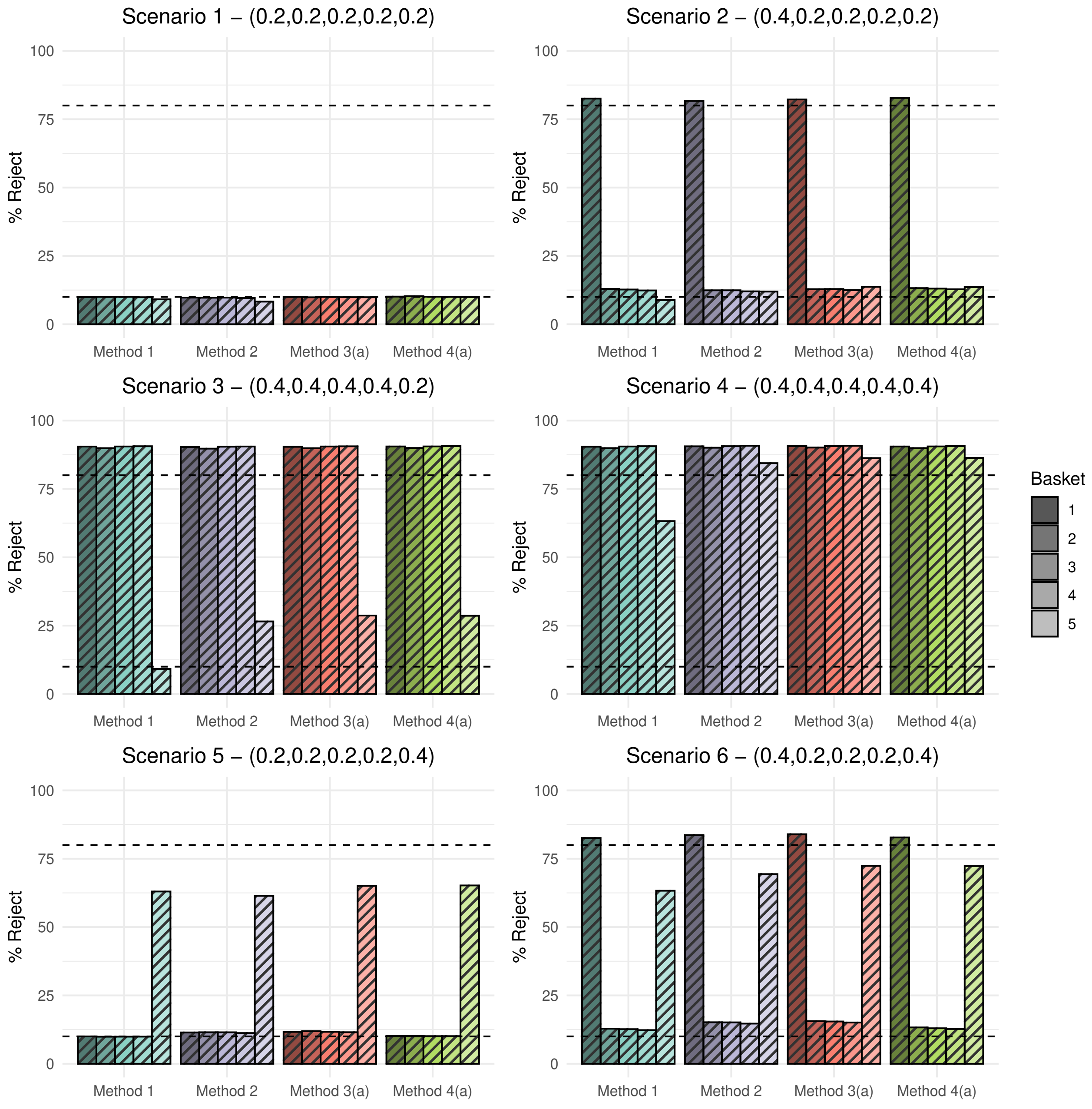

Figure S4: Fixed scenario simulation study results: The percentage of data sets within which the null hypothesis was rejected, where Δ was calibrated under the null to achieve a 10% type I error rate on average. This is plotted for each of the four approaches for adding a basket for all five baskets.

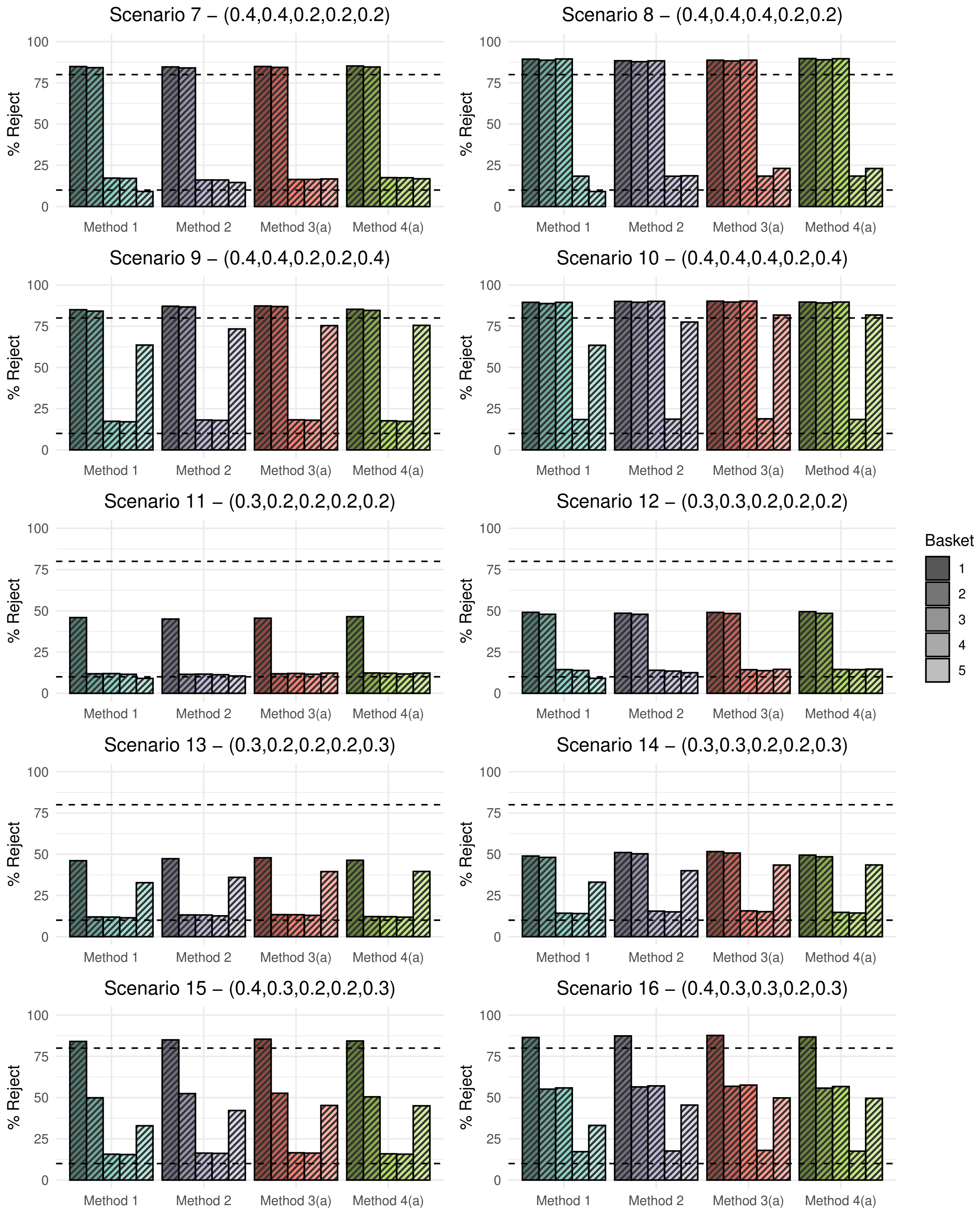

Figure S5: Fixed scenario simulation study results: The percentage of data sets within which the null hypothesis was rejected, where Δ was calibrated under the null to achieve a 10% type I error rate on average. This is plotted for each of the four approaches for adding a basket for all five baskets.

7 Random Scenario Simulation

Results of pair-wise comparisons between approaches for the simulation study presented in the random truth simulation section of the main text. Figures S6 and S7 present these pair-wise comparisons split into existing and new baskets respectively. Table S11 displays full results for all 12 simulation study settings.

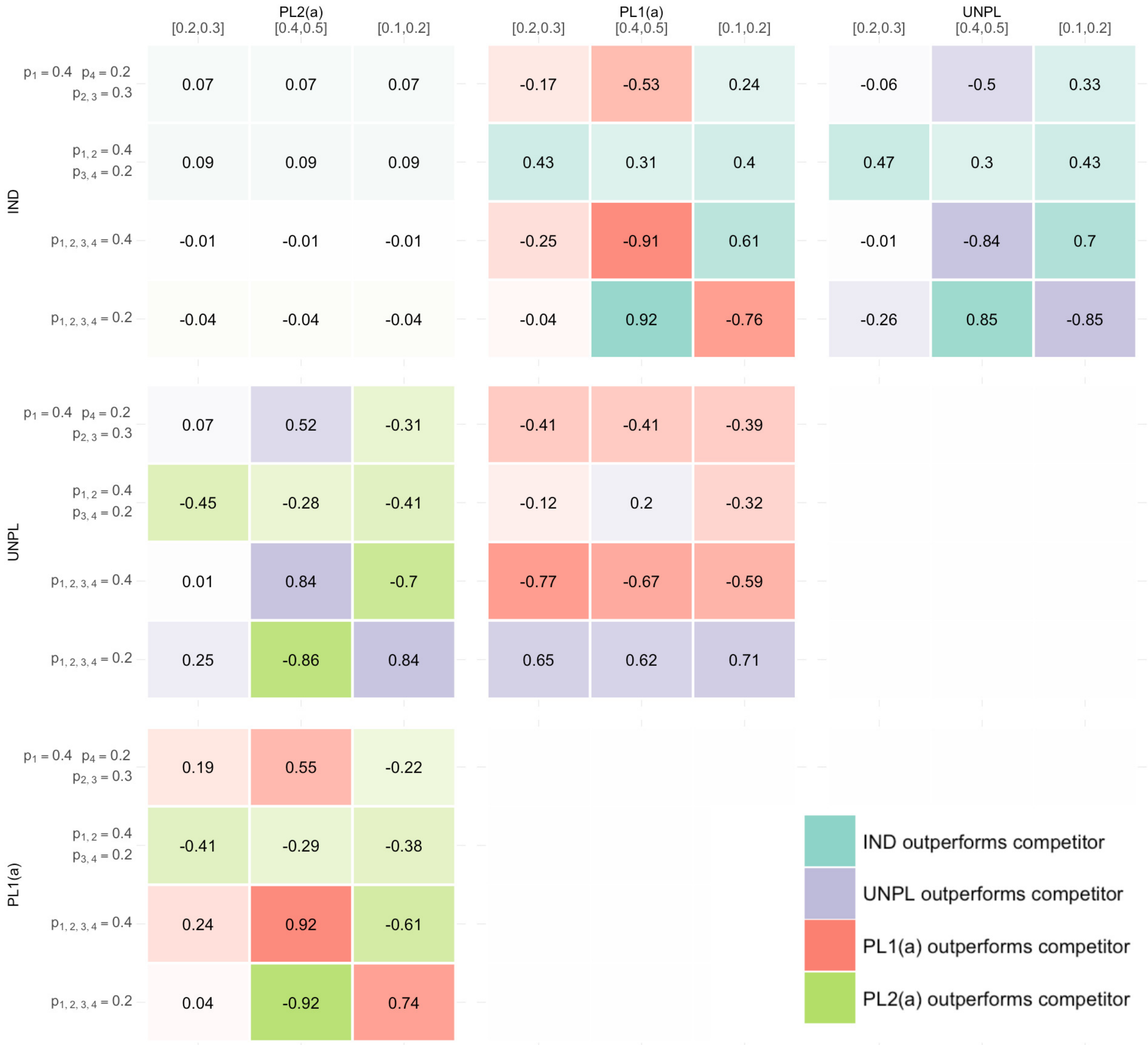

Figure S6: Pair-wise comparison between approaches in each of the 12 simulation settings within which the true response rate in the new basket is varied. The heat map presents the difference in proportion of times the approach corresponding to row gave a correct conclusion over the approach corresponding to column when discrepancies between the two approaches arise in existing baskets only.

First consider just existing baskets and the pair-wise comparisons. Note that IND and PL2(a) are equivalent in these baskets, hence values in such a comparison are centred around 0 with slight simulation error. Results for other comparisons are akin to those presented in the main text, indicating that the driving force behind the results presented in the paper are the difference in proportion of correct conclusion when discrepancies lie in existing baskets.

Then looking at pair-wise comparisons in just the singular new basket, in this case PL1(a) and PL2(a) are equivalent and so results are centred around 0 but with rather a lot of simulation noise. In the comparison between IND

and UNPL, some cases result in all correct conclusions occurring for just one of the two approaches in discrepancies. For example, in the case where homogeneity between new and existing baskets with all having a null response rate, UNPL in which information is borrowed between all baskets leads to correct conclusions in all 309 cases of discrepancies. Whilst in the case of heterogeneity when the existing baskets are effective with the new basket ineffective, IND where the new is analysed independently leads to the correct conclusion in all 40 discrepancies. The number of cases where UNPL outperforms IND differs when looking at just the new basket compared to all discrepancies, with simulations in which the new basket is ineffective now often preferring UNPL.

Much more substantial differences are observed in the comparison between UNPL and PL1(a) under just the new basket compared to overall discrepancies. Previously, in all cases bar when the existing baskets are all null, PL1(a) outperformed UNPL, i.e. a planned addition is preferred to unplanned. However, when considering just the new basket this reverses with UNPL now only preferred when the new basket is ineffective. This arises from the more conservative $\Delta_{k'}$ cut-off under UNPL compared to PL1(a). Note that in most cases very few discrepancies between conclusions under both approaches arise. For instance, when all existing baskets are effective no discrepancies arise when the new basket is ineffective and only 1 or 2 discrepancies arising when it is either marginally effective or effective.

Similar comparisons between UNPL and PL2(a) can be drawn as between UNPL and PL1(a) with cases in which UNPL outperforms PL1(a) also resulting in a conclusions that UNPL outperformed PL2(a). Pair-wise comparisons between IND and PL1(a) approaches and IND and PL2(a) approaches result in the same conclusions as those made in the main text.

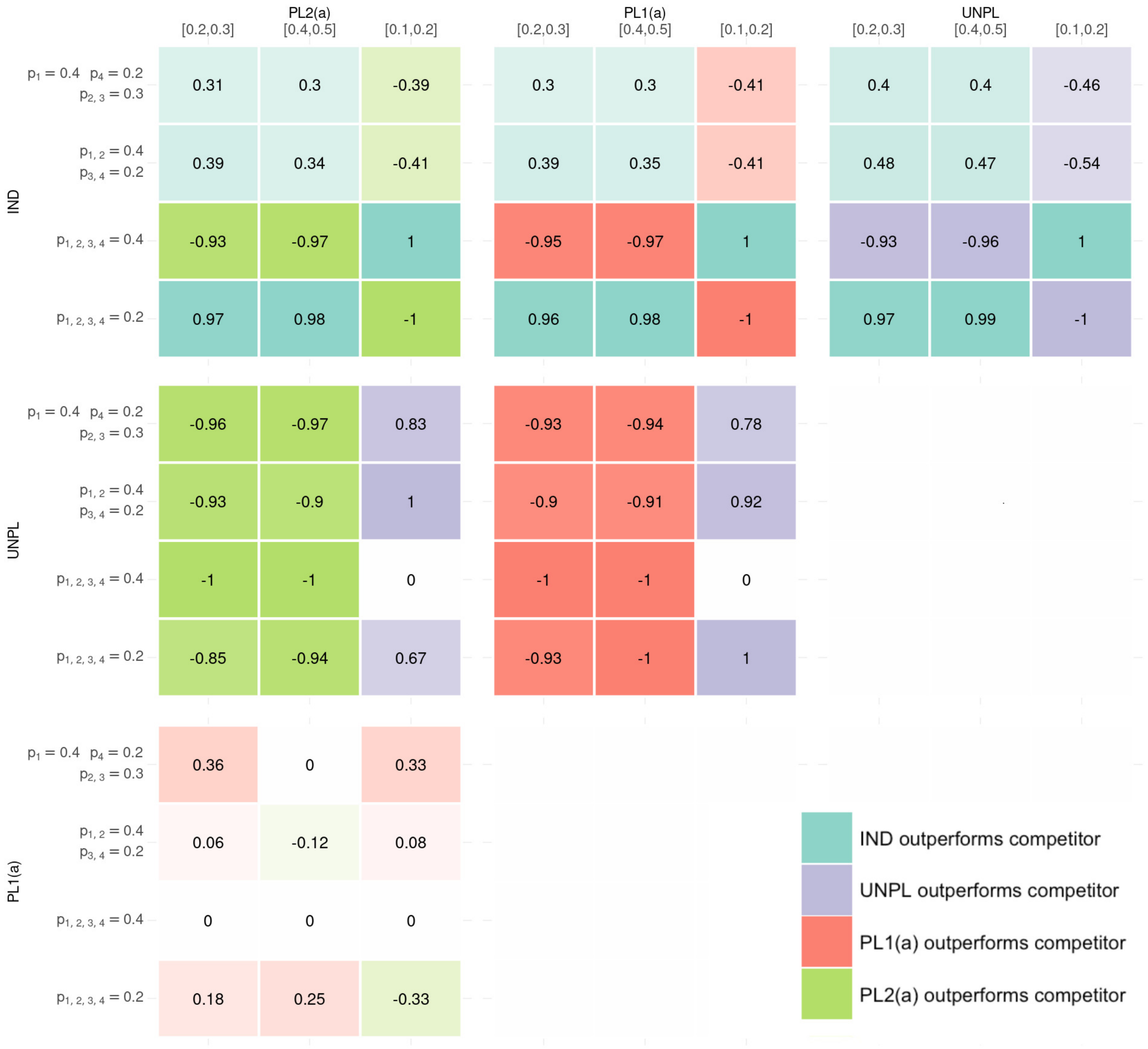

Figure S7: Pair-wise comparison between approaches in each of the 12 simulation settings within which the true response rate in the new basket is varied. The heat map presents the difference in proportion of times the approach corresponding to row gave a correct conclusion over the approach corresponding to column when discrepancies between the two approaches arise in the new basket only.

Table S11: Overall error rates and power for the varied truth simulation study in which the truth in the new basket is varied with the response rate in existing baskets fixed.

	% Reject					FWER	% All Correct
**Setting 1(a)**	**0.2**	**0.2**	**0.2**	**0.2**	**[0.2,0.3]**		
IND	6.40	6.39	6.29	6.35	24.33	21.09	19.17
UNPL	5.94	6.09	5.99	6.19	12.97	19.96	8.79
PL1(a)	6.16	6.39	6.23	6.44	13.23	20.83	8.76
PL2(a)	6.36	6.41	6.29	6.33	13.19	21.12	9.49
**Setting 1(b)**	**0.2**	**0.2**	**0.2**	**0.2**	**[0.4,0.5]**		
IND	6.40	6.39	6.29	6.35	80.73	21.08	63.45
UNPL	7.45	7.33	7.57	7.43	66.96	24.76	48.18
PL1(a)	7.67	7.52	7.77	7.65	67.33	25.43	47.88
PL2(a)	6.36	6.41	6.29	6.33	67.29	21.12	51.67
**Setting 1(c)**	**0.2**	**0.2**	**0.2**	**0.2**	**[0.1,0.2]**		
IND	6.40	6.39	6.29	6.35	5.11	24.95	75.05
UNPL	5.45	5.34	5.29	5.33	2.02	19.05	80.95
PL1(a)	5.58	5.60	5.57	5.50	2.07	19.76	80.24
PL2(a)	6.36	6.41	6.29	6.33	2.06	22.41	77.59
**Setting 2(a)**	**0.4**	**0.4**	**0.4**	**0.4**	**[0.2,0.3]**		
IND	88.86	86.00	86.81	86.87	24.33	0.02	15.24
UNPL	86.74	86.17	86.85	86.91	25.75	0.03	17.35
PL1(a)	87.24	86.82	87.44	87.54	25.76	0.03	17.51
PL2(a)	86.86	86.02	86.79	86.90	25.76	0.03	16.03
**Setting 2(b)**	**0.4**	**0.4**	**0.4**	**0.4**	**[0.4,0.5]**		
IND	86.86	86.00	86.81	86.87	80.72	0.00	49.60
UNPL	88.73	88.26	88.92	88.85	82.49	0.00	53.85
PL1(a)	89.10	88.64	89.16	89.31	82.51	0.00	54.27
PL2(a)	86.86	86.02	86.79	86.90	82.51	0.00	50.58
**Setting 2(c)**	**0.4**	**0.4**	**0.4**	**0.4**	**[0.1,0.2]**		
IND	86.86	86.00	86.81	86.87	5.11	5.11	58.09
UNPL	84.50	83.80	84.59	84.65	5.51	5.51	50.19
PL1(a)	84.86	84.33	85.02	85.21	5.51	5.51	51.76
PL2(a)	86.86	86.02	86.79	86.90	5.51	5.51	57.86
**Setting 3(a)**	**0.4**	**0.4**	**0.2**	**0.2**	**[0.2,0.3]**		
IND	80.88	80.16	10.10	9.88	24.33	17.61	13.19
UNPL	80.02	79.26	10.79	10.48	22.24	18.82	12.08
PL1(a)	80.34	79.75	11.08	10.76	22.81	19.29	12.30
PL2(a)	80.87	80.15	10.20	9.88	22.79	17.68	13.37
**Setting 3(b)**	**0.4**	**0.4**	**0.2**	**0.2**	**[0.4,0.5]**		
IND	80.88	80.16	10.10	9.88	80.72	17.59	43.16
UNPL	81.75	81.06	11.92	11.79	78.25	20.75	40.82
PL1(a)	82.04	81.30	12.35	12.32	79.08	21.59	40.72
PL2(a)	80.87	80.15	10.20	9.88	29.15	17.66	43.58
**Setting 3(c)**	**0.4**	**0.4**	**0.2**	**0.2**	**[0.1,0.2]**		
IND	80.88	80.16	10.10	9.88	5.11	21.81	50.96
UNPL	78.84	77.95	9.62	9.25	4.47	20.55	48.73
PL1(a)	79.40	78.47	9.80	9.50	4.70	21.01	49.13
PL2(a)	80.87	80.15	10.20	9.88	4.71	21.44	50.91
**Setting 4(a)**	**0.4**	**0.3**	**0.3**	**0.2**	**[0.2,0.3]**		
IND	80.	86.00	86.81	86.87	24.33	11.76	3.44
UNPL	80.80	44.94	44.94	12.18	22.76	12.21	4.89
PL1(a)	81.18	45.38	45.52	12.52	23.32	12.55	5.12
PL2(a)	80.77	44.42	44.57	11.89	23.22	11.92	3.69
**Setting 4(b)**	**0.4**	**0.3**	**0.3**	**0.2**	**[0.4,0.5]**		
IND	80.91	44.39	44.46	11.74	80.72	11.74	10.95
UNPL	82.42	47.06	47.63	13.90	78.88	13.90	14.40
PL1(a)	82.81	47.66	48.22	14.26	79.46	14.26	15.13
PL2(a)	80.77	44.42	44.57	11.89	23.22	11.92	3.69
**Setting 4(c)**	**0.4**	**0.3**	**0.3**	**0.2**	**[0.1,0.2]**		
IND	80.91	44.39	44.46	11.74	80.72	11.74	10.95
UNPL	82.42	47.06	47.63	13.90	78.88	13.90	14.40
PL1(a)	82.81	47.66	48.22	14.26	79.46	14.26	15.13
PL2(a)	80.77	44.42	44.57	11.89	79.46	11.89	11.11

8 Investigating the Robustness to the Timing of Addition

In previous simulation studies it is assumed that the timing of addition of a new basket is known prior to the trial, however, this could easily not be the case. This section explores the effect of timing of addition on the performance of analysis methods. Timing of addition is explored by varying the sample size in the new basket. Baskets added early in the trial have a larger sample size than those added at a later time point.

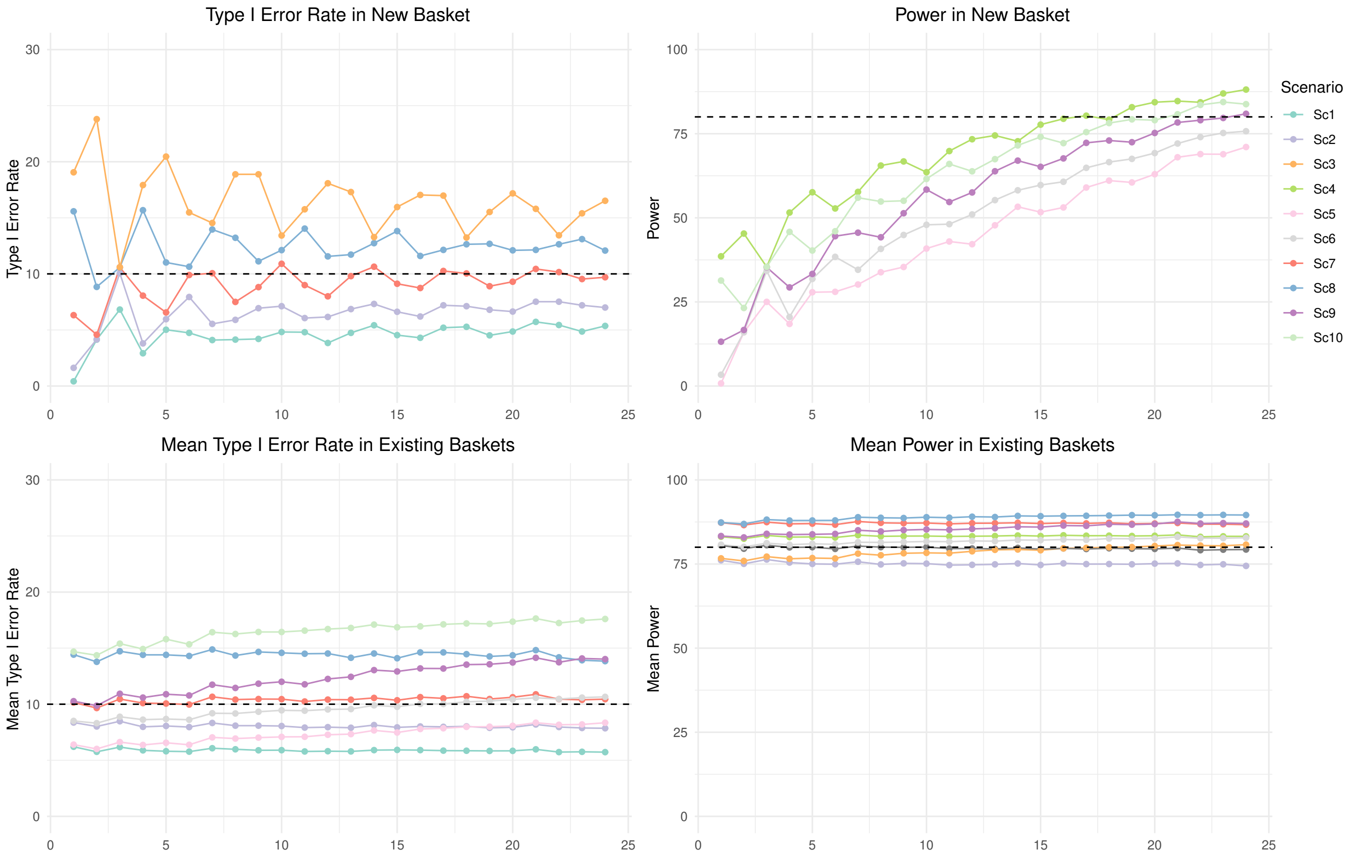

Figure S8: Type I error rate and power under each sample size of n_5 from 1 to 24 by applying PL1(b), split by existing and new baskets.

When a basket is added later in the trial sample sizes may be smaller. This small basket sample size will result in a lack of power and precision of treatment effect estimates. Borrowing information from the larger existing baskets will prove more beneficial in such a setting compared to when the new basket is larger and of similar size to existing baskets. Thus, methods that utilize information borrowing in the new basket become increasingly superior over the IND approach in which an independent analysis is conducted. Improvements in performance in the new basket can still be obtained via information borrowing regardless of timing of addition, however, it is those added later in the trial that will benefit more substantially due to their reduced sample size. Both PL1(a) and PL2(a) make a planned addition of a basket whilst utilizing information borrowing so timing of addition is taken into account in both the calibration process and analysis. In the case that the sample size of the new baskets is unknown, performance of these approaches is more liable to change and thus the robustness of the approaches to the timing of addition is now explored.

To do so, again consider the fixed data scenario simulations setting with four existing and 1 new basket calibrated using the RCaP. In the previous simulation study, sample size of the new basket is assumed as known, consisting of $n_5 = 14$ patients, whilst existing baskets had $n_{k_0} = 24$ patients in each. Now assume n_5 is unknown.

First consider PL1(b) applied to all possible sample sizes from $n_5 = 1$ up to the full sample size of existing baskets, $n_5 = 24$, with separate calibrations of Δ_{k_0} and $\Delta_{k'}$ conducted for each value of n_5. Figure S8 presents the type I error rate and power in new and existing baskets for each value of $n_5 = 1, \ldots, 24$ under scenarios 1-10 as presented in Table S2.

Error rates and power for existing baskets are fairly consistent across all sample sizes, implying the time of addition of a new basket has little to no impact on the performance in these existing baskets, obviously an ideal

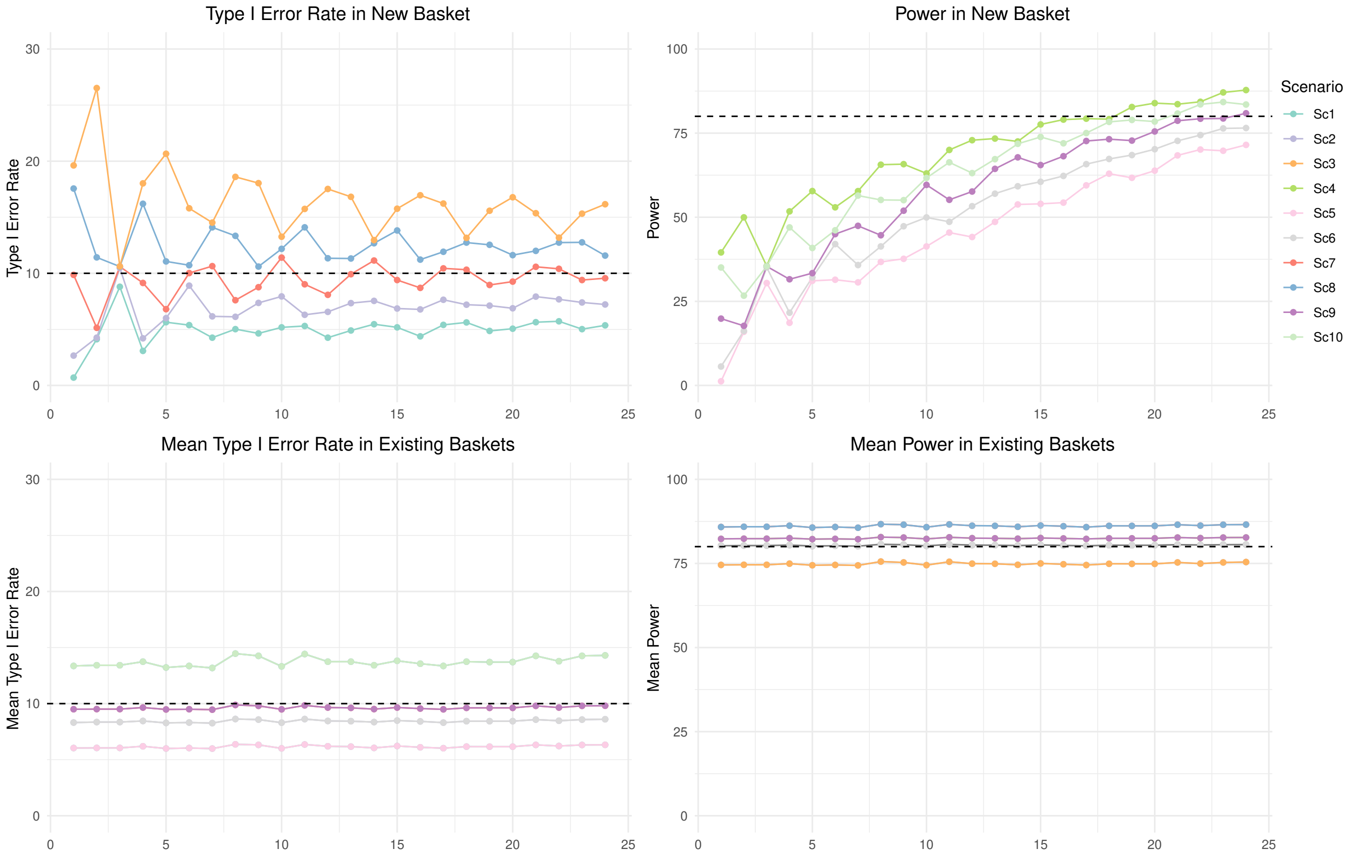

Figure S9: Type I error rate and power under each sample size of n_5 from 1 to 24 by applying PL2(b), split by existing and new baskets.

characteristic.

However, much more noticeable changes are observed in the new basket. Cyclic fluctuations occur in both power and error rate due to the discreetness of data. As expected, power in the new basket generally increases with the sample size as more information is available. Across scenarios, power increases as the number of effective existing baskets also increases. The targeted 80% level is only reached under scenario 6 when $n_5 = 18$ and $n_5 = 21$ under scenario 7. The nominal level is never achieved under scenarios 7 and 8 in which there are none or just one effective existing basket.

In terms of error rates, more variation tends to occur when sample sizes are small, with the greatest error occurring when there are just 2 patients in the new basket (type I error rate of 23.8%) under scenario 5. As the number of effective existing baskets increases, the error rates are uniformly higher, with scenario 5 as the 'worst case' scenario where the only ineffective basket is the new basket. However, there is no general increase or decrease as the sample size increases and thus one cannot make the conclusion that any one sample size results in a more detrimental performance, at least in terms of the type I error rate.

The same interpretations are drawn when looking at the timing of addition under PL2(b) as plotted in Figure S9. As PL1(b) and PL2(b) are equivalent for the new basket, with information borrowed between all baskets, the plots for type I error rate and power in the new basket are identical to that in Figure S8. Again, little to no variation is present in error and power for existing baskets as n_5 changes.

In summary, operating characteristics for PL1(b) and PL2(b) are fairly robust to the timing of addition of the new basket, particularly in the case of existing baskets, with little to no changes in power and error rate with the variation of sample sizes in the new basket. Power in the new basket is obviously improved as the sample size increases but no increase/decrease outside of the cyclic behaviour is observed in error rates, implying the type I error rate will be fairly unaffected by the timing of addition.

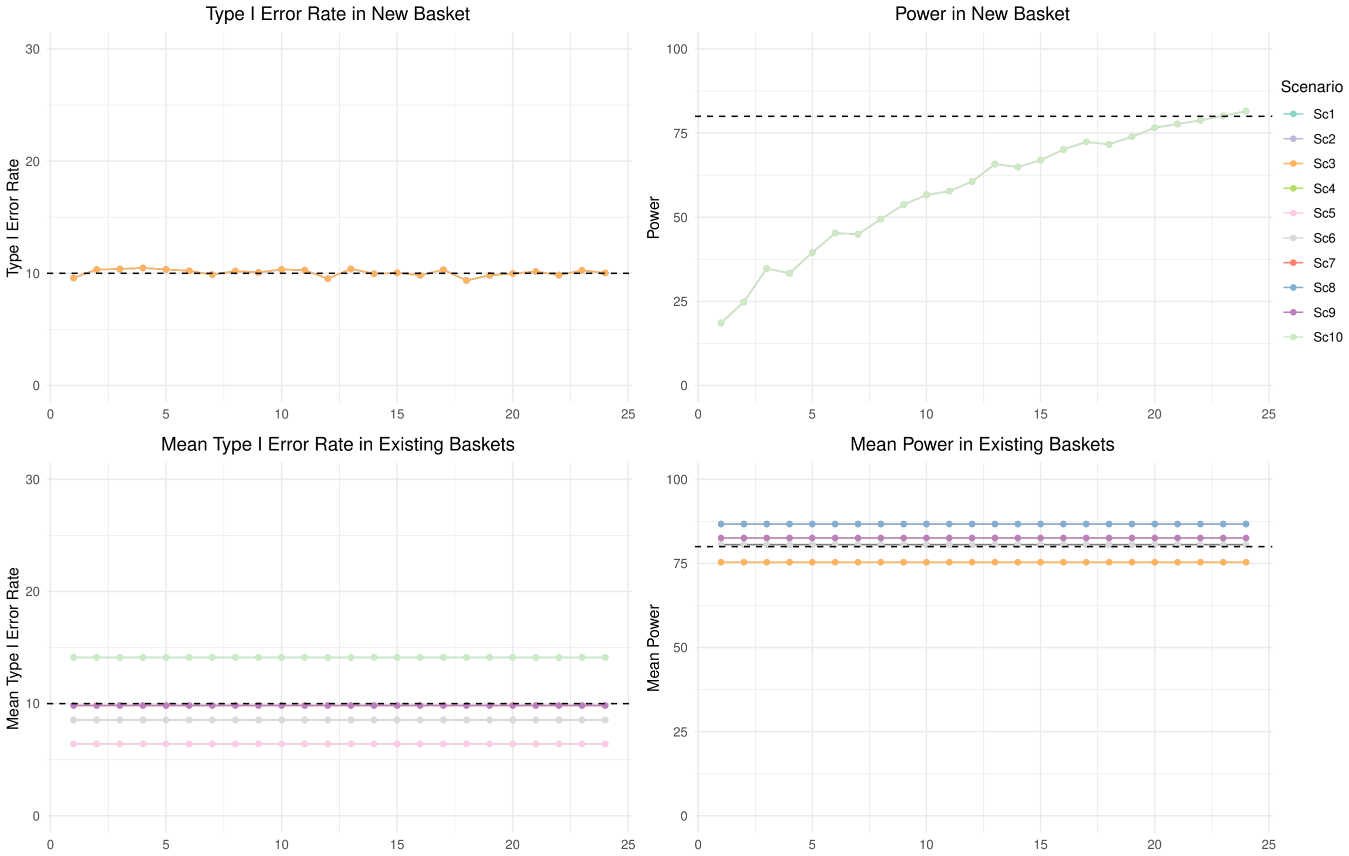

Figure S10: Type I error rate and power under each sample size of n_5 from 1 to 24 by applying IND, split by existing and new baskets.

Under IND, timing of addition will not have an impact on existing baskets due to the independent analysis of new baskets. Thus, when sample sizes are smaller the only effect will be reduced power in the new basket with increased power as sample sizes grow. Under UNPL, the addition is not planned and so timing of addition has no relevance to the calibration procedure.

Figure S10 presents the change in type I error rate and power as the sample size in the new basket varies, split by new and existing baskets for an IND approach. As the new basket is analysed independently, the impact of its sample size on existing baskets is non-existent but also, as each sample size is calibrated to achieve 10% type I error rate, the impact of change in n_5 on error in the new basket is also null. The only variation is in power in the new basket, with larger sample sizes obviously improving power due to the increased certainty in posterior distributions from the added volume of information obtained.

Now under UNPL, the new basket is an unplanned addition and thus the sample size of new baskets has no influence on the calibration procedure. Figure S11 again presents change in type I error rate and power as n_5 varies. Results again imply the sample size in the new baskets has little to no impact on the performance in existing baskets with fairly consistent type I error rates and power across all n_5 values. Power in the new basket increases with the sample size as expected and type I error rates form a cyclic pattern due to the discreetness of data. No general increase or decrease in type I error rate is observed as the sample size changes.

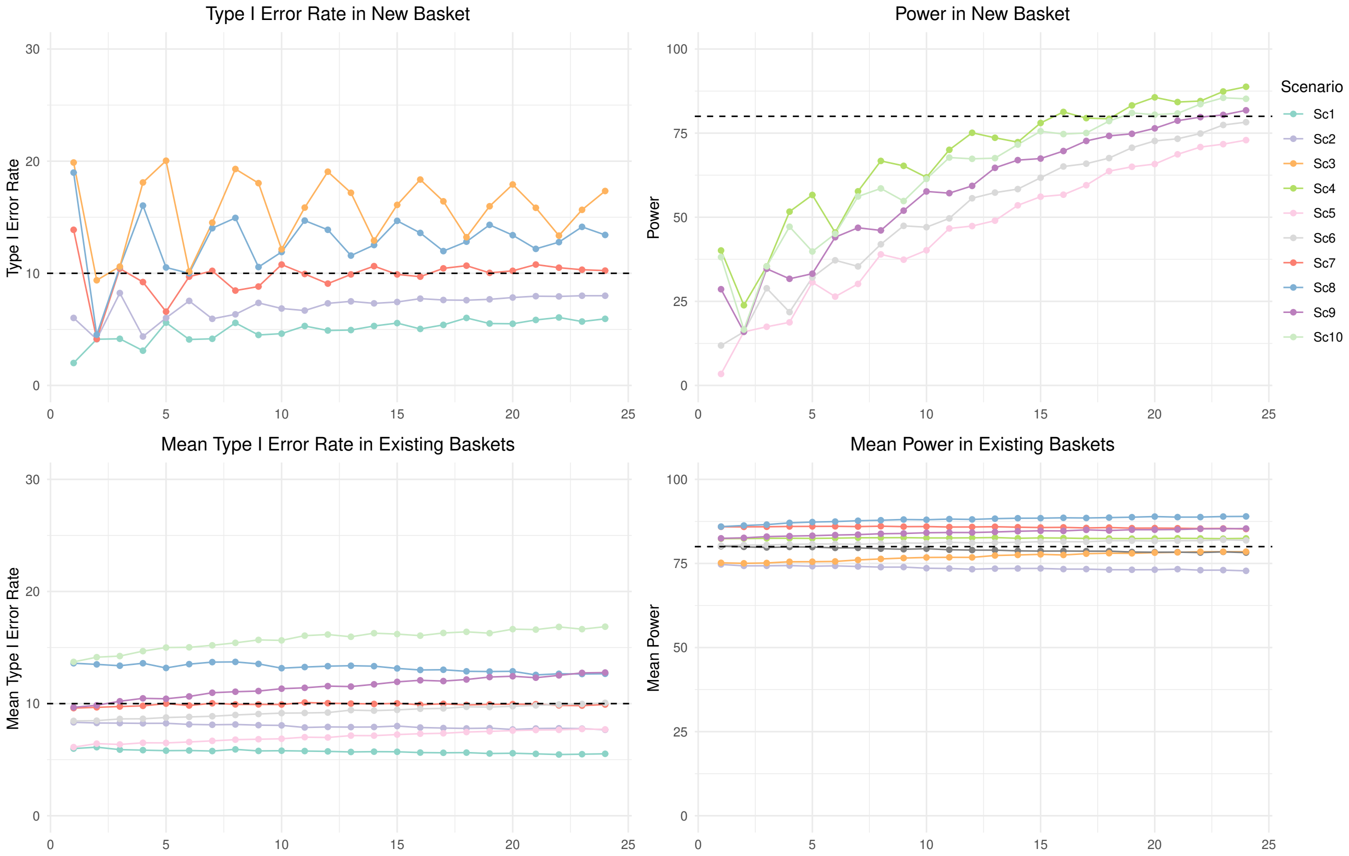

Figure S11: Type I error rate and power under each sample size of n_5 from 1 to 24 by applying UNPL, split by existing and new baskets.

9 Simulation Study - 2 Existing Baskets with 2 New Baskets Added

All simulation studies conducted so far consisted of four existing baskets opening the trial with one additional basket added during the duration. Instead we now consider a case in which there are two baskets starting the trial with a further two baskets added at a later point.

Table S12: Simulation study scenarios

	p_1	p_2	p_3	p_4
Scenario 1	0.2	0.2	0.2	0.2
Scenario 2	0.4	0.2	0.2	0.2
Scenario 3	0.4	0.4	0.2	0.2
Scenario 4	0.4	0.4	0.4	0.2
Scenario 5	0.2	0.2	0.4	0.2
Scenario 6	0.4	0.2	0.4	0.2
Scenario 7	0.2	0.2	0.4	0.4
Scenario 8	0.4	0.2	0.4	0.4
Scenario 9	0.4	0.4	0.4	0.4

The same design parameters as previously implemented are used here with a null and target response rate of $q_0 = 0.2$ and $q_1 = 0.4$ and a sample size of $n_{k_0} = 24$ in existing baskets and $n_{k'} = 14$ in newly added baskets. Models are specified as outlined in Section 1 and data scenarios considered are provided in Table S12. Cut-off values Δ_{k_0} and $\Delta_{k'}$ under the IND, PL1(a) and PL2(a) approaches, are calibrated across scenarios 1-8 with Δ_{k_0} taken as the quantile of posterior probabilities across scenarios 1-2 and 5-8 for basket 2 and $\Delta_{k'}$ as the quantile across scenarios 1-6 of basket 4. For UNPL, the cut-off value is calibrated across just two scenarios: $p = (0.2, 0.2)$ and $p = (0.4, 0.2)$.

Now, as multiple baskets are added during the trial, the IND approach gives two options: (a) analyse both new baskets as independent of existing baskets and one another or (b) analyse both new baskets as independent of existing baskets but borrow from each other using a second EXNEX model.

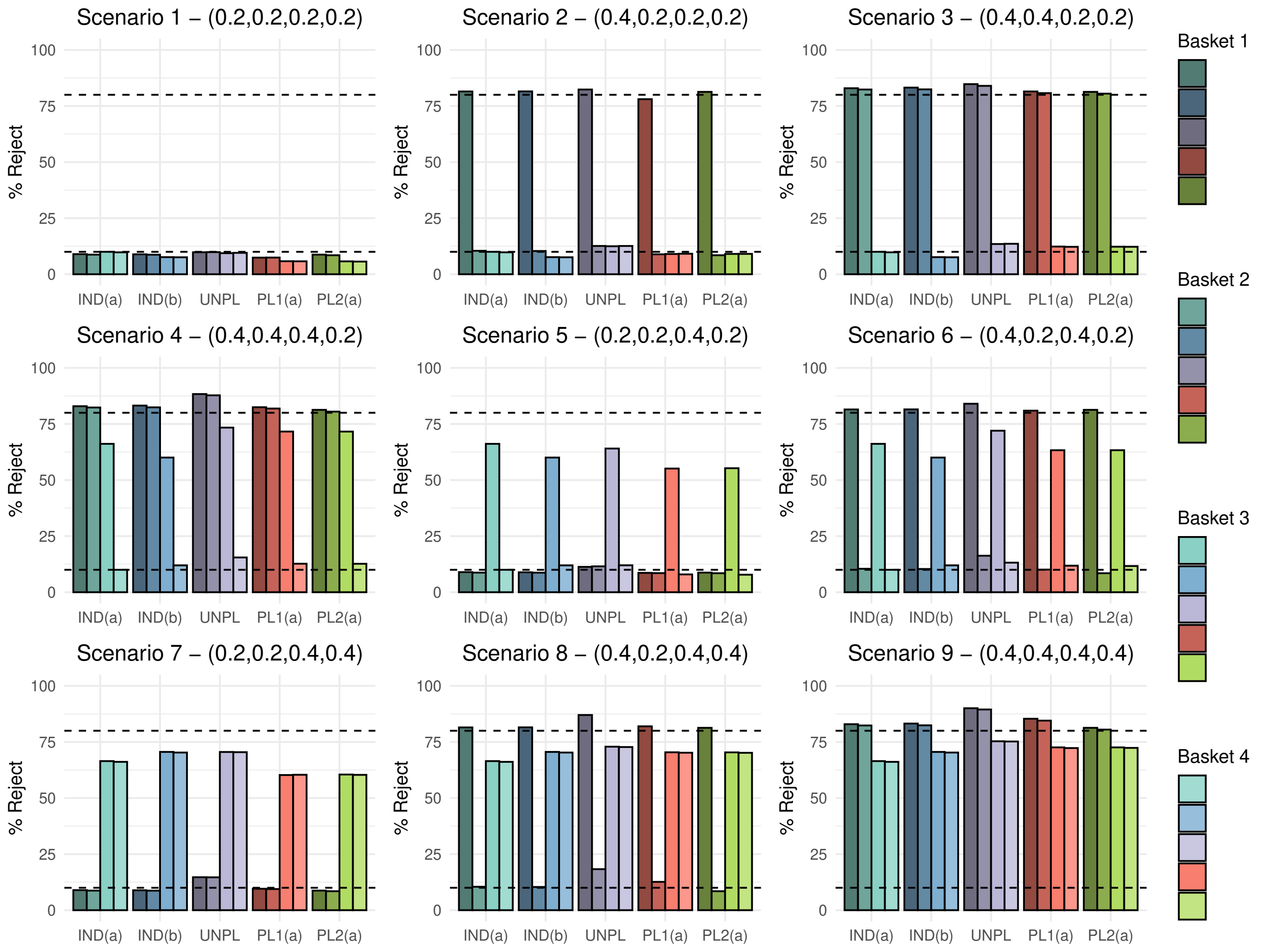

Figure S12: Percentage of data sets within which the null hypothesis was rejected for a simulation study consisting of 2 existing baskets with 2 additional baskets added part-way through the study.

Results for the percentage of data sets within which the null hypothesis was rejected, i.e. type I error rate and power, are presented in Figure S12. First, consider the differences between IND(a) and IND(b). Under IND(a), due to independent analysis, error rates in the new basket are always controlled to the 10% level but the same does not hold for IND(b) in which an EXNEX model allows borrowing between both new baskets. In cases where both new baskets are heterogeneous, this leads to reduced error rates lying below the nominal level (e.g. 7.6% under scenario 1) but in cases of heterogeneity where one new basket is effective and the other ineffective, error rates inflate to approximately 12%. In these scenarios, power is pulled down from 66.2% to 60.1% when utilizing information borrowing. However, significant power can be gained over an independent analysis in cases where both new baskets are effective to treatment (scenarios 7-9). Under IND(a) this power is 66.2% compared to IND(b) with power 70.6%.

Under UNPL, $\Delta_{k_0} = \Delta_{k'} = 0.865$ compared to 0.900 under PL1(a). This reduced cut-off value leads to less conservative rejections under both new and existing baskets. This results in higher power across all cases, with UNPL giving highest power in all scenarios (e.g. UNPL has a power of 89.8% and 75.3% for existing and new baskets respectively under data scenario 9, whereas, PL1(a) has power 84.9% and 72.4% respectively). With this, UNPL also possesses the greatest error inflation up to 18.3%.

Approaches PL1(a) and PL2(a) are equivalent for the new baskets so results differ only in existing baskets. Some cases with more noticeable differences are scenario 2 in which power is increased significantly under PL2(a) at 81.3% compared to 78.1% under PL1 with indistinguishable difference in error; scenario 8 in which both approaches give

similar power but PL1 has higher error rates at 12.6% compared to PL2(a) at 8.5% and finally, scenario 9 in which power in existing baskets is greater under PL1(a) at 84.8% compared to 80.9% under PL2(a).

To conclude, results in this simulation study present fairly similar results to the previous case consisting of 4 existing and one new basket. One of the main differences lies in the UNPL approach which has a far less conservative cut-off than any of the other approaches. This occurs because there are only 2 existing baskets that can be used to calibrate UNPL and as such, estimates lack certainty and only 2 data scenarios are calibrated across. Also displayed in this case, is the potential losses one can make when utilizing IND(b) in all cases bar when both baskets are homogeneous to treatment.
